# A Functional Screen Reveals an Extensive Layer of Transcriptional and Splicing Control Underlying RAS/MAPK Signaling in Drosophila

**DOI:** 10.1371/journal.pbio.1001809

**Published:** 2014-03-18

**Authors:** Dariel Ashton-Beaucage, Christian M. Udell, Patrick Gendron, Malha Sahmi, Martin Lefrançois, Caroline Baril, Anne-Sophie Guenier, Jean Duchaine, Daniel Lamarre, Sébastien Lemieux, Marc Therrien

**Affiliations:** 1 Institute for Research in Immunology and Cancer, Laboratory of Intracellular Signaling, Université de Montréal, Montréal, Québec, Canada; 2 Département de médecine, Université de Montréal, Montréal, Québec, Canada; 3 Département d'informatique et de recherche opérationnelle, Université de Montréal, Montréal, Québec, Canada; 4 Département de pathologie et de biologie cellulaire, Université de Montréal, Montréal, Québec, Canada; Baylor College of Medicine, United States of America

## Abstract

The small GTPase RAS is among the most prevalent oncogenes. The evolutionarily conserved RAF-MEK-MAPK module that lies downstream of RAS is one of the main conduits through which RAS transmits proliferative signals in normal and cancer cells. Genetic and biochemical studies conducted over the last two decades uncovered a small set of factors regulating RAS/MAPK signaling. Interestingly, most of these were found to control RAF activation, thus suggesting a central regulatory role for this event. Whether additional factors are required at this level or further downstream remains an open question. To obtain a comprehensive view of the elements functionally linked to the RAS/MAPK cascade, we used a quantitative assay in Drosophila S2 cells to conduct a genome-wide RNAi screen for factors impacting RAS-mediated MAPK activation. The screen led to the identification of 101 validated hits, including most of the previously known factors associated to this pathway. Epistasis experiments were then carried out on individual candidates to determine their position relative to core pathway components. While this revealed several new factors acting at different steps along the pathway—including a new protein complex modulating RAF activation—we found that most hits unexpectedly work downstream of MEK and specifically influence MAPK expression. These hits mainly consist of constitutive splicing factors and thereby suggest that splicing plays a specific role in establishing MAPK levels. We further characterized two representative members of this group and surprisingly found that they act by regulating mapk alternative splicing. This study provides an unprecedented assessment of the factors modulating RAS/MAPK signaling in Drosophila. In addition, it suggests that pathway output does not solely rely on classical signaling events, such as those controlling RAF activation, but also on the regulation of MAPK levels. Finally, it indicates that core splicing components can also specifically impact alternative splicing.

## Abstract 2

A global RNAi screening approach in Drosophila cells identifies a large group of transcription and splicing factors that modulate RAS/MAPK signaling by altering the expression of MAPK.

## Author Summary

The RAS/MAPK pathway is a cornerstone of the cell proliferation signaling apparatus. It has a notable involvement in cancer as mutations in the components of the pathway are associated with aberrant proliferation. Previous work has focused predominantly on post-translational regulation of RAS/MAPK signaling such that a large and intricate network of factors is now known to act on core pathway components. However, regulation at the pre-translational level has not been examined nearly as extensively and is comparatively poorly understood. In this study, we used an unbiased and global screening approach to survey the Drosophila genome—using Drosophila cultured cells—for novel regulators of this pathway. Surprisingly, a majority of our hits were associated to either transcription or mRNA splicing. We used a series of secondary screening assays to determine which part of the RAS/MAPK pathway these candidates target. We found that these factors were not equally distributed along the pathway, but rather converged predominantly on *mapk* mRNA expression and processing. Our findings raise the intriguing possibility that regulation of *mapk* transcript production is a key step for a diverse set of regulatory inputs, and may play an important part in RAS/MAPK signaling dynamics.

## Introduction

The RAS/MAPK pathway consists of a core module of three kinases (RAF, MEK, and ERK/MAPK) that transmit signals downstream of the small GTPase RAS. Upstream factors such as receptor tyrosine kinases (RTKs), which respond to extracellular signals, lead to RAS activation by a guanine nucleotide exchange factor (GEF). GTP-loaded RAS then triggers the sequential activation of RAF, MEK, and MAPK; active RAF phosphorylates and activates MEK, which in turn phosphorylates and activates MAPK [1]. Unlike RAF and MEK, MAPK has a variety of cytoplasmic and nuclear substrates that include transcription factors such as c-Jun, c-Fos, p53, ELK1, c-Myc, c-Myb, STAT1/3, SRF, and SMAD1/2/3/4 [2]–[4]. Phosphorylation of these targets, and others, by MAPK induces a wide range of cellular responses that include proliferation, differentiation, and survival [5]. Also, RAS/MAPK signaling's important role in oncogenesis and various developmental disorders has been recognized early on and abundantly studied [6],[7].

Over the last two decades, genetic screens in metazoan models such as *Drosophila* and *Caenorhabditis elegans* have been instrumental in identifying a growing list of key regulators of the RAS/MAPK pathway such as *sos* [8], *csw* [9], *ksr* [10]–[12], *Cbl* [13], *dos* [14], *mts*/*PP2A* [15], *sur-8*/*soc-2* [16],[17], *cnk* [18], *spry* [19], *sur-6* [20], *PTP-ER* [21], *let-7* [22], *alph*/*PP2C* [23], and *hyp*/*ave* [24],[25]. Thus, these studies and research conducted in other systems have revealed a large network of factors whose regulatory activity converges on the core MAPK module [1],[5],[6],[26],[27]. This regulatory network includes complex features such as feedback loops [28]–[30], compartmentalization [1],[31], crosstalk with other signaling pathways [32], allosteric modulation via dimerization [27],[33], and the formation of larger order complexes called nanoclusters [34]. While the function of the core module is well characterized, many aspects of the network that surround it are still poorly understood, including its protein composition. Also, many of the identified regulators influence RAS-mediated RAF activation, which is in agreement with the fact that this particular step is subjected to a tight and complex regulation [27]. In comparison, fewer positively acting components have been found to act downstream of RAF, suggesting that MEK and MAPK activation depends on more simple regulatory mechanisms. Alternatively, such modulators might have eluded detection. Finally, most of the regulatory input that has been described so far acts at the post-translational level. Comparatively little is known on how RAS/MAPK component expression is controlled.

The success of the aforementioned genetic screens typically relied on the qualitative modification of a visible phenotype. This consideration, together with the technical limitations associated with genetic screening procedures, usually limit results to a handful of confirmed hits. RNA interference (RNAi) used as a functional genomics tool provides the possibility of a more comprehensive type of analysis providing a systematic means to functionally annotate the genome [35],[36]. Moreover, the possibility of using quantitative assays, in particular, allows for the identification of a much wider range of regulators [37]. However, the considerable number of candidates often identified by this methodology has made the perspective of rapid functional annotation a daunting task.

Here, we present the results of a genome-wide RNAi screen in *Drosophila* S2 cells that specifically focused on signal regulation between RAS and MAPK. Validated hits were submitted to a series of secondary assays aimed at positioning their regulatory input with respect to the three core kinases. In addition to identifying and correctly positioning most of the components previously known to mediate RAS-induced MAPK activation, the screen led to the discovery of several new factors that act at different steps along the pathway. Notably, we identified five novel components that act upstream of RAF. The homologs of these five proteins are part of a complex named striatin-interacting phosphatase and kinase complex (STRIPAK) [38] that also includes PP2A, which is known to regulate RAF activation [39],[40]. Unexpectedly, the majority of our candidates did not map to the interval between RAS and RAF, but were instead positioned further downstream. These included some transcription factors that we found regulate the transcript abundance of *mek*, *mapk*, or of the MAPK phosphatase *PTP-ER*. However, most of the novel factors were associated with mRNA processing and were found to act downstream of MEK and to regulate *mapk* splicing. Among these were components of the exon junction complex (EJC), which we and others have previously reported to be involved in regulating the splicing of the *mapk* pre-mRNA [41],[42]. In particular, depletion of the EJC was found to alter the splicing of *mapk*'s long introns and cause a reduction in the amount of functional protein product. In this study, we focus on the function of a larger group of canonical splicing factors that also regulate *mapk* splicing. We show that the impact of these factors on alternative splicing (AS) of *mapk* differs from what we previously described for the EJC, indicating that two different types of regulatory input act on this step in *mapk* expression.

Thus, in addition to providing a comprehensive view of regulatory factors influencing signal transmission between RAS and MAPK, this work suggests that pathway output does not solely rely on post translational regulatory events, such as those controlling RAF activation, but is also tightly governed by the regulation of the expression of core components. In particular, the expression of MAPK emerges as a focal point for multiple different regulatory inputs.

## Results

### Identification of New RAS/MAPK Pathway Regulators

To systematically search for and categorize new factors that specifically modulate signaling between RAS and MAPK, we employed a screening strategy that involved three distinct steps: (1) a primary genome-wide RNAi screen, (2) a validation screening step aimed at eliminating false positives, and (3) validated candidates were submitted to a series of a secondary screens to establish the position of their regulatory input relative to known pathway components (epistasis) and assess their specificity to RAS/MAPK signaling (Figure S1A).

**Figure 1:**
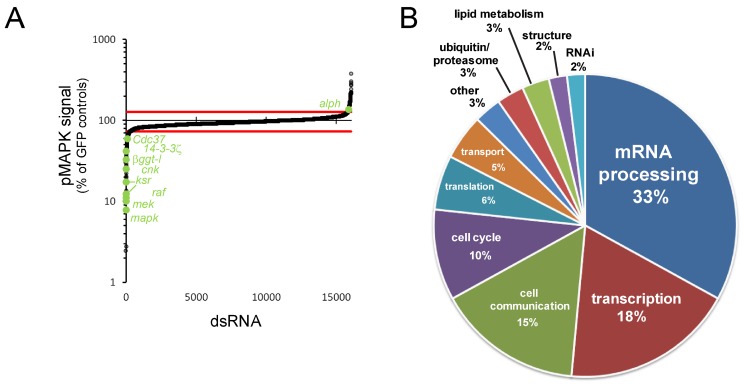
Primary screen results. (A) pMAPK signal of primary screen dsRNA probes normalized to *GFP* dsRNA-treated controls. A majority of expected pathway regulators were identified as hits (labeled in green) outside of the cutoff margins (red lines). (B) Functional distribution of validated hits, based on GO term annotation of *Drosophila* genes or their predicted homologs.

We employed an automated immunofluorescence-based microscopy assay that quantitatively detected variations of dually phosphorylated MAPK (pMAPK) in Drosophila S2 cells. This assay was used to screen a genome-wide long double-stranded RNA (dsRNA) library for modulation of pMAPK levels induced by RAS^V12^ expression (Figure S1B and S1C). The results from this primary screen and all subsequent screens are made available online at the IRIC RNAi database (http://www.bioinfo.iric.ca/iricrnai). 309 hit genes, which reproducibly altered pMAPK signal, were identified in the primary screen (Table S1). Importantly, core RAS/MAPK pathway components (e.g., *raf/phl*, *mek/Dsor1*, *mapk/rl*, *cnk*, and *ksr*) were amongst the strongest hits that decreased the pMAPK signal (Figure 1A; Table S1). Other known positively acting genes were also identified, such as *14-3-3ζ* and the RAF chaperone *Cdc37* [43]. Another expected hit was *βggt-I*, which encodes a factor involved in RAS prenylation [10],[44],[45]. Also expected, the PP2C phosphatase *alph* was identified as a negative regulator [23].

We next conducted two successive validation steps to address readily identifiable sources of false positives, namely effects on the *pMet-RAS^V12^* expression system and dsRNA off-target effects. 101 genes of the initial 309 primary hits passed both validation criteria (Figure S2; Table S1; Text S1). Validated genes were then assigned to broad functional categories on the basis of their associated gene ontology (GO) terms and on the functions of predicted homologs. Interestingly, transcription and mRNA processing factors composed, together, roughly half of our candidates (Figure 1B), and mRNA processing was the most highly enriched GO term of our hit set (Table S2). Despite the fact that mRNA splicing factors are often enriched in RNAi screen hit lists [46], we chose not to apply a selection bias against any group of genes at this stage. Therefore, all of the candidate genes passing both primary and validation screen criteria were evaluated in secondary screens without distinction.

### Secondary Screens: Epistasis

To further characterize the 101 candidate genes, we conducted a series of secondary screens that can be subdivided into three groups: (1) MAPK activation induced by stimuli upstream of RAS, (2) epistasis screens involving MAPK activation at the level of or downstream of RAS, and (3) JNK activation screens aimed at addressing specificity to the MAPK pathway context (Figure S3; detailed results in Tables S3, S4, S5).

Four distinct MAPK activation assays using RTKs or *GAP* RNAi (Figure S3) were conducted to assess the degree to which pathway activity could be perturbed by depletion of the candidate genes in different activation contexts occurring upstream of RAS. Although a few exceptions were found, in most cases, we observed signal modulation that was generally consistent with our RAS^V12^ results.

**Figure 2:**
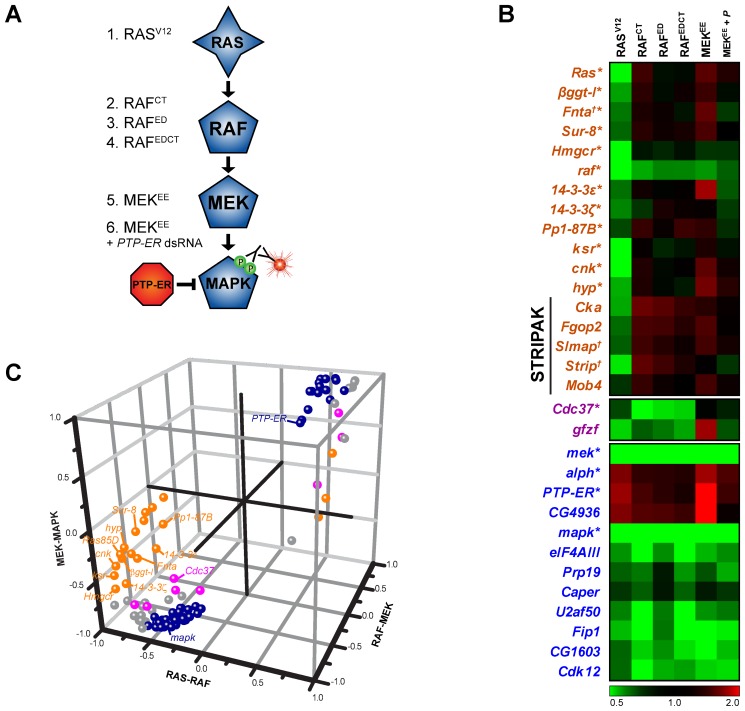
Epistasis analysis. (A) MAPK pathway models depicting the secondary screen assays used to conduct the epistasis analysis. See Text S1 for full secondary assay information. (B) Epistasis screen results are shown for *bona fide* MAPK pathway components as well as a selection of candidates. Results are presented as pMAPK values normalized to *GFP* dsRNA treated controls. *Bona fide *MAPK pathway components are identified with an asterisk (*). Genes to which we have associated new gene symbols are marked with a cross (†). (C) Known pathway components (labeled) and experimental candidates are assigned to specific epistasis intervals. The calculated Pearson correlation *r* between the epistasis screen data profiles and the three predetermined profiles for epistasis intervals (see Methods) are represented on three axes (*x*-axis, RAS-RAF; *y*-axis, MEK-MAPK; *z*-axis, RAF-MEK). Values near 0 represent poor correlation while values near 1 (negative regulators) or −1 (positive regulators) indicate high correlation with a given epistasis profile. Candidates were assigned to the RAS-RAF (orange), RAF-MEK (magenta), or MEK-MAPK (dark blue) interval on the basis of the highest distance value. Candidates that could not be assigned to a specific interval (distance values within [−0.5, 0.5]) are shown in grey. Detailed epistasis screen results are available in Table S3.

Next, epistasis experiments were carried out using S2 cell lines expressing either constitutively activated forms of RAF or MEK (Figures 2 and S3). The aim of these experiments was to position the identified genes in relation to the core kinases of the pathway by comparing the values from the RAS, RAF, and MEK activation assays. To do this, we calculated the correlation of our screening data with theoretical profiles of hypothetical components acting within three possible epistasis intervals (RAS-RAF, RAF-MEK, and MEK-MAPK) using an uncentered Pearson's correlation metric (Figure 2C; Table S3; Text S1). All known pathway components were positioned correctly by this approach. For example, *Ras85D*, *ksr*, *cnk*, *hyp/ave*, *14-3-3ζ*, *14-3-3ε*, and *βggt-I* are part of a group of genes that suppressed RAS^V12^, but not activated RAF or activated MEK; these components were thus correctly positioned in the RAS-RAF interval (Figure 2B and 2C). Nine additional genes also fell into this category (Table S3) and therefore represent potentially novel pathway regulators acting at this level. Strikingly, while only eight hits (including the RAF chaperone Cdc37) mapped between RAF and MEK, most of the candidates (69) were assigned to the MEK-MAPK interval (Table S3), with the majority of these being factors not previously linked to RAS/MAPK signaling. This unexpected finding suggests that additional regulatory events that escaped prior detection are lying downstream of MEK.

RAC1^V12^ and peptidoglycan (PGN) were then used as stimuli in two JNK activation assays as a proxy to evaluate specificity to the RAS/MAPK signaling context (Figure S3). Very few of the candidates modulated pJNK to a similar extent as they did pMAPK (Table S4). One of these was the ALPH PP2C phosphatase, whose depletion increased both pMAPK and pJNK signals. This is consistent with our recent findings demonstrating that ALPH negatively regulates both MAPK and JNK signaling [23],[47]. Remarkably, the vast majority of the RNA processing factors identified in the primary screen did not modulate pJNK levels and thereby argued for their specific role in RAS/MAPK signaling (Table S4).

### Predicted Protein Complexes Have Similar Functional Profiles

**Figure 3:**
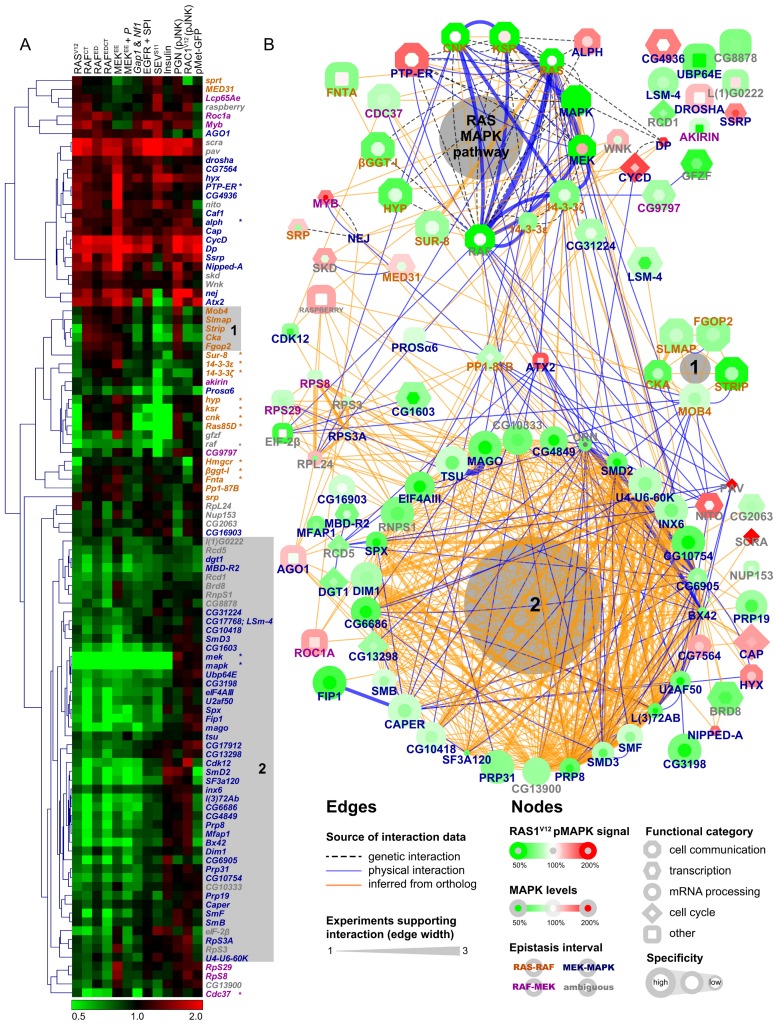
Predicted protein complexes have similar secondary screen functional profiles. (A) Unsupervised hierarchical clustering of secondary screen results. The 13 secondary assays are listed at the top of the clustering diagram. *Bona fide* MAPK pathway components are identified with an asterisk. (B) Protein interaction network (PIN) assembled using interaction data from *Drosophila* and homologs from other species. Edge color represents the source of the interaction data and edge width denotes the number of distinct experimental evidences for a given interaction. The coloring of the node border represents RAS^V12^ screen results while the coloring of the node center reflects MAPK protein levels. Node shapes reflect the functional category of the hits and specificity results are represented by the size of nodes. In both panels, epistasis results are represented by the coloring of gene symbols in orange (RAS-RAF), magenta (RAF-MEK), dark blue (MEK-MAPK), or grey (ambiguous). MAPK pathway components, STRIPAK (shaded area 1) and splicing factors (shaded area 2) group together in both the clustering analysis and PIN. Note that the full name is used for the gene raspberry, instead of *ras*, to avoid confusion with *Ras85D*.

We next submitted our secondary screen data to unsupervised hierarchical clustering to group candidates with similar profiles together (Figure 3A). *Bona fide* pathway components with similar functions are clearly grouped together by this analysis. For example, *Ras85D*, *ksr*, *cnk*, and *hyp* all act at the level of RAF activation and all show very similar profiles. Both 14-3-3 isoforms, which also act at this level, are grouped together and are also close to the first group of genes involved in RAF activation, as is *βggt-I*, a component involved in RAS prenylation. On the basis of these findings, we can expect that candidates who have a similar profile to *bona fide* RAS/MAPK pathway components might in fact share the same function as these components.

Following this, we sought to identify putative protein complexes as well as related factors in our set of candidates by constructing a protein interaction network (PIN) based on publicly available protein and genetic interaction data (Figure 3B). The canonical RAS/MAPK components are clearly grouped together in this network and at least two other complexes can be clearly distinguished. The first consists of components of the STRIPAK complex and the second is composed primarily of mRNA processing factors. Remarkably, the components of both complexes also group together in similar functional profiles in our clustering analysis (groups “1” and “2” in both panels of Figure 3).

### Regulation of RAS/MAPK Pathway Gene Expression and Specificity

**Figure 4:**
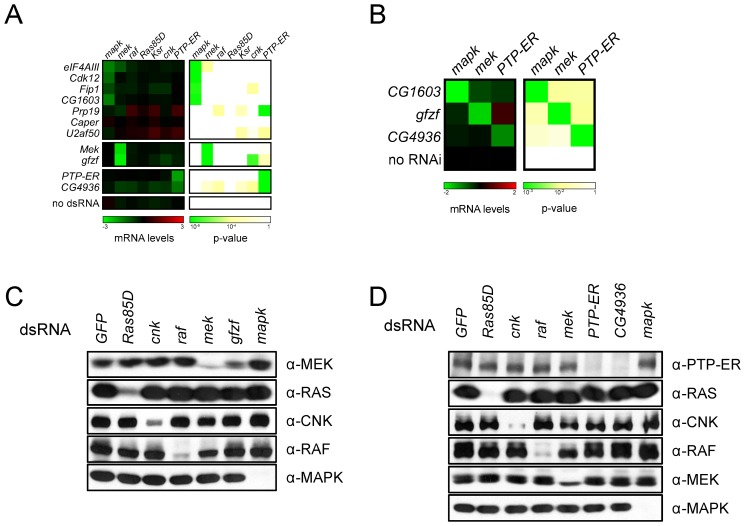
Screen candidates modify the expression of RAS/MAPK components. (A) The transcript levels of RAS/MAPK components are altered by the depletion of some candidates. All candidates were tested in an initial qPCR secondary screen; the results shown here are from a separate qPCR confirmation experiment (see Text S1). The left panel shows transcript levels for the RAS/MAPK components listed on the top following treatment with the indicated dsRNAs (labels to the left). mRNA levels are expressed as log_2_ ratios of *GFP* dsRNA treated controls. The right panel shows the associated *p*-values (unpaired two-tailed Student's *t*-test). dsRNA targeting *gfzf* had a similar effect to the *mek* dsRNA with a −2.86 reduction in *mek* transcript levels (*p*-value, 4.2×10^−8^). *CG4936* dsRNA caused a −1.37 reduction in *PTP-ER* transcript levels (*p*-value, 1.2×10^−8^), which was slightly weaker than the −1.88 reduction (*p*-value, 3.0×10^−9^) measured for the *PTP-ER* dsRNA. *Cdk12*, *Fip1*, and *CG1603* dsRNAs behaved similarly to *eIF4AIII* dsRNA, used as a control for *mapk* transcript depletion, with *mapk* transcript levels <−0.75 and a *p*-value<1×10^−4^. (B) *In vivo* RNAi experiments confirm cell culture qPCR results. Hairpin RNAi constructs were expressed in larvae under the control of a heat shock-inducible flip-out actin promoter. qPCR experiments were performed on L3 eye disc lysates. mRNA levels are expressed as log_2_ ratios of a no RNAi control (flies carrying the flip-out promoter without a RNAi construct). Results were similar to those in cell culture qPCR experiments in (A): *CG1603* RNAi caused a reduction in *mapk* levels (−2.06; *p*-value, 5.3×10^−4^), *gfzf* RNAi reduced *mek* levels (−1.66; *p*-value, 5.4×10^−4^), and *CG4936* reduced *PTP-ER* levels (−1.06; *p*-value, 5.7×10^−4^). (C and D) The levels of RAS/MAPK pathway components in S2 cells were evaluated by Western blot with the indicated antibodies (labels to right of panel) following treatment with the indicated dsRNA reagents (top labels). (C) Mirroring the qPCR data, a specific depletion of MEK levels was observed in *gfzf* dsRNA treated cells. (D) Also in agreement with the qPCR data, a specific decrease in PTP-ER levels was observed upon depletion of *CG4936*.

Given that several of our candidate genes are linked to RNA processing and transcription, we hypothesized that these factors might be acting on the expression of one or multiple RAS/MAPK pathway components. We first investigated the impact of our candidates on the expression of core RAS/MAPK pathway component transcripts (*Ras85D*, *raf*, *mek*, *mapk*, *ksr*, *cnk*, and *PTP-ER*) by quantitative PCR (qPCR) (Figure S4A; Tables S5 and S8). A specific effect on the *mapk* transcript was observed upon depletion of *mago* and *eIF4AIII* (Figure 4A; Tables S5 and S6) as we have previously reported [41]. At least three other factors (*Cdk12*, *Fip1*, and *CG1603*) also seemed to modulate the transcript levels of *mapk*. Moreover, two factors, *gfzf* and *CG4936*, were found to modulate the levels of *mek* and *PTP-ER*, respectively (Figure 4A; Table S6). *CG1603*, *gfzf*, and *CG4936* were subsequently tested in larval eye disc tissue where similar results were obtained (Figure 4B).

Surprisingly, aside from the candidates mentioned above, most hits did not appear to cause a significant change in the transcript levels of pathway components. We had previously observed that qPCR assays targeting *mapk* are not always strongly affected by the splicing changes induced by EJC depletion. On the other hand, a reverse transcription PCR (RT-PCR) assay spanning the whole *mapk* transcript is a more sensitive tool allowing for detection of small splicing changes [41]. Based on this premise, and on the fact that almost all the splicing factors we identified mapped downstream of MEK, we decided to systematically examine the impact of these factors on *mapk* splicing. Consequently, we used the RT-PCR assay that had been used with the EJC to examine *mapk* splicing. Interestingly, not only did this experiment reveal that nearly all the splicing factors in our set caused shifts in the *mapk* RT-PCR profile, but these RT-PCR profiles were also clearly different from those produced by EJC depletion (Figure S5). Thus, while the impact of most of our candidates on *mapk* expression may not be apparent when measuring total transcript abundance, a clear impact on the different *mapk* isoforms can be observed by RT-PCR, indicating that these factors regulate AS of *mapk*.

**Figure 5:**
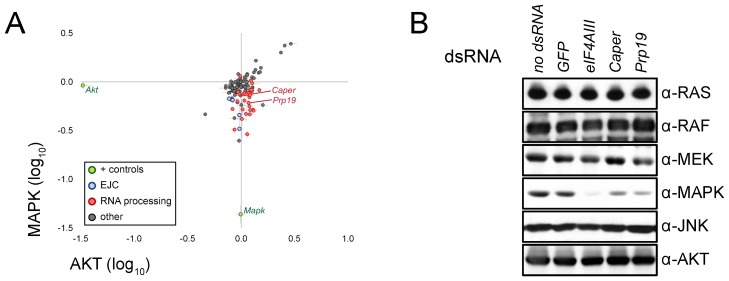
Splicing factors cause a decrease in MAPK protein levels. (A) Candidates were tested in an immunofluorescence-based secondary screen to evaluate their impact on MAPK protein levels. The results from this experiment show that RNA processing factors (red) cause a reduction in MAPK levels without impacting AKT (used as a negative control). This impact is similar to that which was observed for EJC components (blue). *mapk* and *Akt* dsRNA positive controls are also shown (green). (B) The specific effect on MAPK levels is confirmed by Western blot for both *Prp19* and *Caper* dsRNA treated samples. The impact on MAPK is similar, albeit slightly weaker, to that observed following *eIF4AIII* depletion. The levels of other MAPK pathway components (RAS, RAF, and MEK) as well as other signaling pathway components (AKT and JNK) did not appear to vary significantly following depletion of these factors.

In the case of the EJC, the splicing changes were accompanied by a corresponding decrease in MAPK protein levels. Because of this decrease, we decided to also measure the impact of our candidates on MAPK protein levels using quantitative immunofluorescence. This analysis confirmed that most of the factors positioned downstream of MEK (including most of the RNA processing factors) also caused a reduction of MAPK protein levels. Conversely, AKT protein levels, which were used as a control, were not generally sensitive to depletion of these same factors (Figure 5A). We also verified the impact on MAPK and two other pathway components (RAS and CNK) by Western blot. Most candidates that caused a reduction in MAPK levels did not impact the levels of RAS, CNK, or AKT (Figure S6), which mirrored the results from the immunofluorescence experiment. Thus, both evaluations of MAPK protein levels agreed with the RT-PCR experiments suggesting that the changes in splicing results in a reduction of MAPK protein abundance.

Most of the other hits, including those positioned at the RAS-RAF and RAF-MEK intervals, did not appear to cause a change in the expression of RAS/MAPK pathway components. However, we observed that a minority of candidates seemed to cause fluctuations in multiple proteins or transcripts. Likewise, these candidates also tended to impact some or all of the non-RAS/MAPK related assays we tested them in (Table S5). Moreover, these hits were also more frequently present in hit lists of other published RNAi screens (Table S4). Consequently, in order to discriminate such non-specific hits from higher quality candidates, we derived a scoring system that factored in results from the pJNK assays, pMet-green fluorescent protein (GFP) expression, hit occurrence in previously published RNAi screens, and the impact on measured protein and transcript levels (see Table S4; Text S1). As expected, a few of the splicing factors in our list had low specificity scores. On the other hand, most of the factors that selectively affected MAPK levels, including the majority of the splicing factors, were not present in previous screen hit lists more frequently than *bona fide* RAS/MAPK factors and generally displayed a good specificity to the RAS/MAPK context (Tables S4 and S5). This finding suggests that discriminating against an entire category of genes on the basis of the enrichment of that category in previous screen hit sets is a strategy that can lead to elimination of meaningful candidates.

### RAS-RAF Candidates: The Usual Suspects and STRIPAK

Most of the positive regulators positioned in the RAS-RAF interval were factors that had previously been linked either to RAS prenylation or regulation of RAF activation. As previously mentioned, one of the RAS prenylation factors we identified was *βggt-I*, which was originally identified in *Drosophila* [10],[45]. In addition to *βggt-I*, two other factors that are known to function in RAS prenylation in other organisms were also identified: *Hmgcr* and *Fnta* (CG2976). FNTA is the alpha farnesyltransferase subunit for mammalian RAS proteins [48]. The hydroxymethylglutaryl-CoA reductase HMGCR functions in the cholesterol biosynthesis pathway and is required for farnesylation of RAS and other membrane-associated proteins [49],[50]. In our Western blot experiments, all three of these factors were observed to cause a mobility shift in RAS suggesting that RAS geranylgeranylation is impaired [51], and thus that these factors act on RAS in *Drosophila* (Figure S6A, S6C, and S6J).

RAF activation is arguably the most tightly regulated step of the MAPK module [1],[27],[52]. Multiple components are involved in a series of events that link up RAF to RAS, anchor RAF at the plasma membrane, allow RAF to adopt and maintain an active conformation and finally enable efficient substrate targeting [52]. Phosphorylation and dephosphorylation events control progression throughout these steps [52]. Of these, the removal of the phosphate moiety on the S346 residue of *Drosophila* RAF (equivalent to S259 of human RAF1) is one of the pivotal regulatory events as it is thought to trigger the release of 14-3-3, which otherwise sequesters RAF in the cytoplasm [52]. Of the factors involved in RAF activation, all of the expected factors (*ksr*, *cnk*, *hyp*, *14-3-3ε*, and *14-3-3ζ*) were correctly positioned at the RAS-RAF interval (Figure 2B). Two other candidates, *Pp1-87B* and *Sur-8*, also clustered together with the set of known factors acting in the RAS-RAF interval. These two factors had not previously been shown to act on MAPK signaling in *Drosophila*, but evidence from other organisms indicates that they might act at this level [16],[17],[40], which is consistent with our results. Of particular interest, one study has found a mammalian complex composed of PP1, SUR-8, and MRAS and linked it to dephosphorylation of the S259 residue on C-RAF [53].

The only other positive regulators in the RAS-RAF epitasis group that were not previously linked to RAS/MAPK signaling were five components that form the smallest of two complexes in our network (CKA, STRIP, SLMAP, FGOP2, and MOB4) (Figure 3B). Three of these components are distantly related to budding yeast alpha factor arrest (FAR) complex components, which are involved in signaling G1 arrest upon alpha factor stimulation [54]. More recently, a protein complex comprising Striatin, the catalytic subunit of PP2A, the STE20 family kinase STK24, and four additional core proteins was identified in human cells and named the STRIPAK complex [38]. The core of this complex was suggested to serve as a protein platform that specifies PP2A and/or STK24 action. Remarkably, the five RAS-RAF proteins identified are homologs of the non-catalytic members that make up the core STRIPAK complex. CKA, which is the fly Striatin homolog, has previously been demonstrated genetically to act as a positive regulator of JNK signaling [55]. MOB4 has also been previously studied genetically in *Drosophila*, where it appears to participate in mitotic spindle assembly [56]. The three other members, STRIP, FGOP2, and SLMAP, have not been extensively studied in flies and are named on the basis of their mammalian counterparts.

Consistent with their ability to work together as a complex, the five STRIPAK homologs had similar effects in all the secondary screens and epitope-tagged variants co-immunoprecipitated in binary co-expression experiments (Figures 3A and S7). Notably, their depletion also suppressed JNK activation induced by RAC1^V12^, suggesting that CKA/Striatin modulates signaling through this pathway and that the other STRIPAK members act in conjunction with Striatin in this context. This is also consistent with the findings that TRAF3-interacting JNK-activating modulator (T3JAM), one of the SLMAP homologs, is linked to JNK signaling [57] and with a recent report that identified *Cka* as a suppressor of JNK signaling [58]. Furthermore, depletion of STRIPAK components reduced pMAPK signal induced by insulin, activated Sevenless RTK (SEV^S11^) and GAP RNAi, but only marginally affected EGFR signaling (Figure S5). This suggests that the role of STRIPAK differs depending on the MAPK and JNK activation contexts.

**Figure 6:**
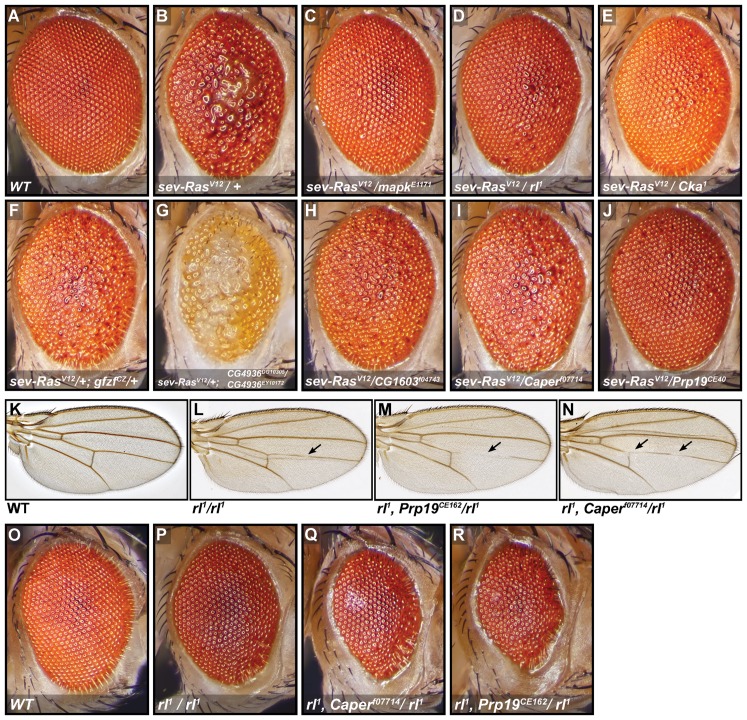
RNAi screen candidates interact genetically with RAS/MAPK pathway components. (A–J) The *Ras^V12^* rough eye phenotype is dominantly suppressed by heterozygous mutations in *Cka*, *gfzf*, *CG1603*, *Fip1*, *Prp19*, *Caper*, and a trans-heterozygous mutation in *CG4936*. Fly eyes of the indicated genotypes were imaged by stereomicroscopy. The *mapk* alleles *mapk^E1171^* and *rl^1^* are used as positive controls. All fly eye images are from female flies except *CG4936^DG10305^*/*CG4936^EY10172^*, which is from a male fly; the rough eye phenotype was observed to be similar in males and females except in this case where males displayed a stronger genetic interaction. (K–N) Genetic interactions with *rl^1^* wing vein deletion phenotypes. *rl^1^*/*rl^1^* flies display a slight deletion of the mid-section of the L4 wing vein that is not fully penetrant. The L4 deletion is enhanced, sometimes extending to the posterior cross vein (pcv) in *Prp19^CE162^* and *Caper^f07714^* heterozygous backgrounds (pictures shown served to illustrate detailed scoring results in Figure S8H). (O–R) Genetic interactions with *rl^1^* rough-eye phenotypes. The weak rough eye phenotype observed in *rl^1^* homozygotes is shown. The severity of this phenotype is increased in heterozygous mutant backgrounds for *Prp19* and *Caper*; these flies display a further decrease in eye size and an increased eye roughness.

To confirm the involvement of STRIPAK complex components in RAS/MAPK signaling *in vivo*, we conducted genetic interaction experiments using *Cka*/Striatin mutant alleles [55]. RAS/MAPK activity is required for neuronal photoreceptor and cone cell differentiation during *Drosophila* eye development [59],[60]. Expression of *Ras^V12^* under the control of the eye specific *sev* promoter/enhancer regulatory sequences produces extra photoreceptor cells, which causes a characteristic rough-eye phenotype (Figure 6B) [61]. This rough eye phenotype was dominantly suppressed in a *Cka* heterozygous mutant background (Figures 6E and S8A). Extra wing vein material produced by a constitutively active *Egfr* allele, *Egfr^Elp^*, was also dominantly suppressed by *Cka* mutant alleles to a degree comparable to a weak loss-of-function allele of *rl*/*mapk* (Figure S8E and S8J). In agreement with these results, wing vein deletions were significantly enhanced in a *shp-2*/*csw* hemyzygous mutant background when a *Cka* mutation was introduced in this context (Figure S8D, S8I, and S8K). Moreover, consistent with the role of STRIPAK in RAS/MAPK signaling, loss-of-function of *Cka* activity impaired R7 photoreceptor cell differentiation, which is a classical RAS/MAPK-dependent developmental event (Figure S9).

In addition to PP2A/*mts*, we noted that some of the other STRIPAK components described in Goudreault and colleagues [38] were not identified in our primary screen. However, one of these, *GckIII*, was one of the validated regulator in the InR-driven MAPK screen reported by Adam Friedman and Norbert Perrimon [62]. This raised the possibility that *GckIII* might have an impact on pathway activity in alternate activation contexts. To address this, we examined the effects of depleting *GckIII* in RAS^V12^, insulin, and *GAP* RNAi assays. We found that while this had little impact on RAS^V12^-induced MAPK activation, *GckIII* depletion had an effect comparable to *Fgop2* depletion in the insulin and *GAP* RNAi contexts (unpublished data). This indicates that *GckIII* may function upstream or in parallel to RAS and raises the intriguing possibility that the STRIPAK complex regulates multiple aspects of the larger RTK/RAS/MAPK pathway.

Since Striatins are defined as PP2A regulatory (B) subunits [63] and STRIPAK was initially described as a PP2A associated complex, it is possible that STRIPAK assumes this role in the context of RAF activation. We observed a modulation of RAS^V12^ signaling upon depletion of the catalytic subunit of PP2A (*mts*), but not of the regulatory B subunit, *tws*, which support the notion that STRIPAK components are functioning as PP2A regulatory (B) subunits in this context (unpublished data). A similar function for STRIPAK has recently been described in the context of Hippo signaling, where it was found to associate with PP2A and HPO [64]. Finally, in agreement with our findings, another recent report has linked the CKA subunit to RAS/MAPK signaling [65] and another study conducted in *Neurospora* has indicated that MAPK regulates STRIPAK function, suggesting the possibility of regulatory feedback phosphorylation [66].

### RAF-MEK Candidates: gfzf

Only 13 of our candidates were found to act downstream of RAF and upstream of MEK. Of these, the RAF chaperone *Cdc37* was the only *bona fide* pathway regulator. Among the others, *CG8878* was of interest as it is the homolog of the mammalian Vaccinia-Related Kinase (VRK) genes of which two (VRK1 and VRK3) have been recently identified in a recent screen for KRAS synthetic-lethal factors [67]. Also, another study has shown that VRK2 interacts with MEK and KSR1, potentially acting on MEK activation [68]. However, in this context, VRK2 acts as a negative regulator whereas *CG8878* appears to act positively on RAS signaling in our experiments.

**Figure 7:**
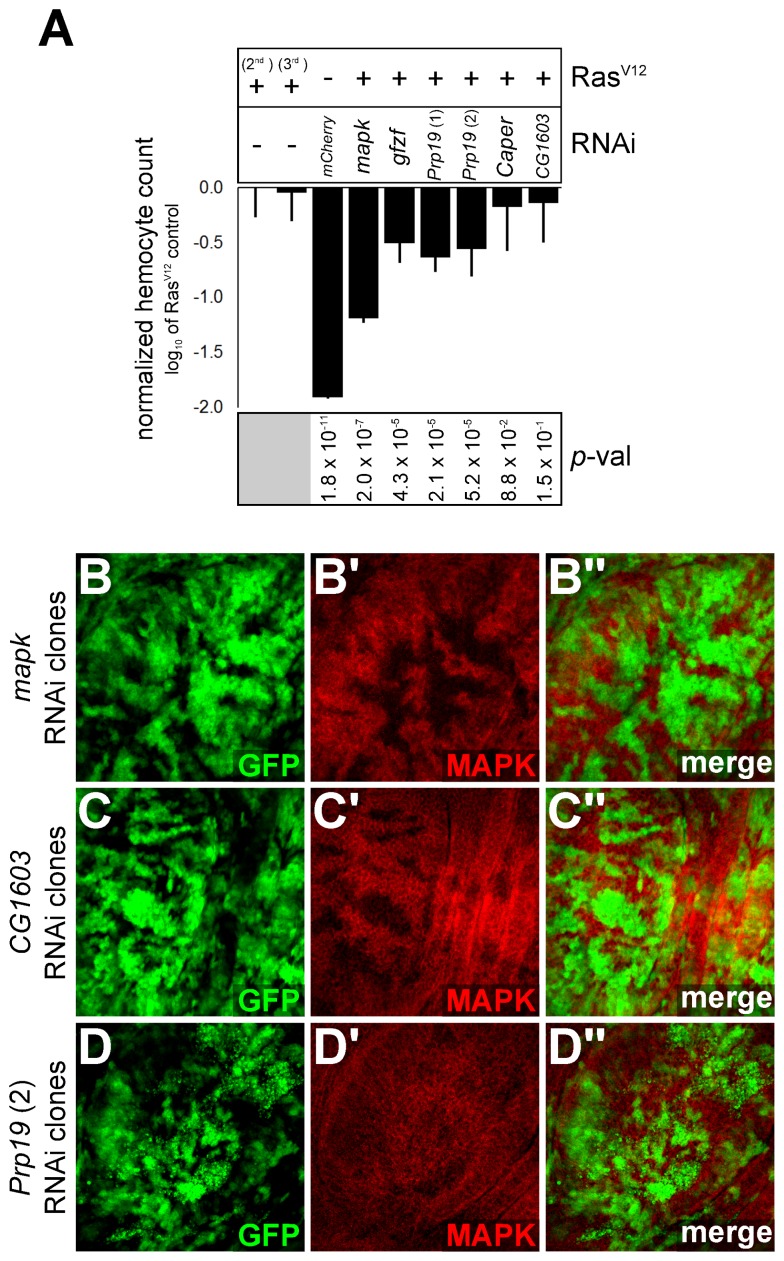
*In vivo* evidence of impact of RAS/MAPK signaling. (A) Impact of candidates on *Ras^V12^*-induced hemocyte proliferation. A *Hemolectin-Gal4* driver was used to co-express RAS^V12^ (on either Chromosome 2 or 3) with the RNAi constructs or a *UAS-lacZ* control. GFP positive hemocytes were counted by automated microscopy. The total hemocyte count is expressed as log_10_ ratio of the *Ras^V12^* control (second chromosome *UAS-Ras^V12^* fly line). Expression of *Ras^V12^* increased hemocyte count by approximately 100-fold compared to a *UAS-mcherry* RNAi negative control without *Ras^V12^*. Co-expression of a *mapk* RNAi with *Ras^V12^* was used as a control for reduced proliferation. A Student's *t*-test (unpaired, two-tailed) was performed comparing candidates to the appropriate *Ras^V12^* control. (B–D) 3rd instar larval imaginal discs showing reduction in MAPK protein levels in GFP positive clones expressing RNAi hairpin constructs. Two different *Prp19* RNAi constructs were tested in wing imaginal discs (one is shown here) and found to produce a slight, but consistent reduction in MAPK levels in the clonal tissue. This was more visible in clones with a stronger GFP signal and was sometimes accompanied by signs of apoptosis (small GFP positive fragments), which are visible in (D).

The most interesting candidate that fell within the RAF-MEK interval was GST-containing FLYWCH zinc-finger protein (*gfzf*). GFZF was initially found because of the property of its GST domain to bind to glutathione sepharose beads [69]. It does not, however, have any clearly identified function assigned to it, though there are indications that it may act as a co-factor for the E2F transcription factor [70]. Also, recent RNAi screens have suggested that *gfzf* may be acting downstream of PDGF to control cell size [71] and as a factor functioning in the G2/M DNA damage checkpoint [72]. In our qPCR experiments, *gfzf* clearly stood out from other candidates as it was the only factor that caused a strong reduction in *mek* transcript levels and had the closest profile to the *mek* RNAi itself (Figures 4A, 4B, and S4A). This observation also extended to the protein product as we observed a clear reduction in MEK levels upon *gfzf* knockdown (Figure 4C). In flies, two different *gfzf* loss of function alleles suppressed the *Ras^V12^* rough eye phenotype (Figures 6F and S8A). *gfzf^cz811^*also increased the severity of wing vein deletions in hemizygous *csw^lf^* males (Figure S8D and S8K). Finally, knocking down *gfzf* reduced RAS^V12^-induced hemocyte proliferation (Figure 7A). Together, these observations suggest *gfzf* is regulating *mek*, possibly by acting as a positive transcription factor. Since FLYWCH domain proteins have been found to negatively control miRNA expression in *C. elegans* [73], one interesting alternative is that *gfzf* represses the production of a miRNA that targets the *mek* transcript.

Another candidate, *CG9797*, was clustered close to *gfzf* through the functional screen data and the qPCR results (Figures 3A and S4A), and its protein product is predicted to interact with GFZF (Figure 3B). In the qPCR screen, *CG9797* knockdown caused a weak reduction in *mek* transcript levels; it is the fourth strongest hit in terms of reducing *mek* levels, after the dsRNAs targeting *gfzf*, *mek*, and *RpL24* (Table S5). From this, and since *CG9797* encodes another zinc finger protein, it is possible that this factor works in conjunction with GFZF as a transcriptional regulator. Recently, the chromatin remodeling factors *Geminin* and *Brahma* have been found to modulate MEK protein expression in drosophila wing discs [74], raising the possibility that *gfzf* might be acting in conjunction with these factors.

### MEK-MAPK Candidates: Multiple Inputs Converge on mapk Splicing

mRNA processing and transcription factors formed the largest group of hits in our study and also the largest complex in our network analysis (Figures 3B and S1B), and almost all these factors mapped to the MEK-MAPK epistasis interval (Table S5). While the majority of the candidates in this category cause changes in *mapk* expression, one clear exception was *CG4936*. This gene encodes a zinc finger protein of unknown function that is distantly related to human ZBTB20, a BCL-6 like transcription factor that is expressed in hematopoietic tissues and lymphoid neoplasms [75]. *CG4936* also mapped downstream of MEK, but was not found to influence *mapk* expression. A first clue as to the function of this factor was provided by the observation that *CG4936* behaved very similarly to *PTP-ER* in our functional screens; of all the candidates, it has the closest profile to *PTP-ER* (Figure 3A). One of the two MEK^EE^-based MAPK activation assays involved the use of *PTP-ER* RNAi to increase the pMAPK signal. Surprisingly, while both *PTP-ER* and *CG4936* RNAi significantly increase MEK^EE^-induced pMAPK levels, combining *CG4936* and *PTP-ER* RNAi did not have an additive effect on pMAPK levels (Figure 2B; Table S5). This finding suggests that these factors work together in the same regulatory pathway. *CG4936* also clustered close to PTP-ER in our qPCR screen (Figure S4A). Consistent with this, both qPCR and protein analysis revealed that *CG4936* knockdown respectively caused a specific reduction in *PTP-ER* transcript and protein levels (Figure 4A, 4B, and 4D). Furthermore, in our genetic interaction experiments, flies trans-heterozygous for two P-element insertion alleles of *CG4936 (CG4936^EY10172^*/*CG4936^DG10305^*) showed an enhancement of the *Ras^V12^* rough eye phenotype (Figure 6G) and suppressed lethality and phenotypes caused by a homozygous *mapk*/*rl^1^* hypomorphic allele (Figure S8C, S8G, and S8H). *CG4936^EY10172^* alone suppressed the lethality and wing vein deletions of *csw^lf^* hemizygous males (Figure S8I and S8K), as did the double *CG4936* mutant (unpublished data). Moreover, *csw^lf^* homozygous females were observed in a background heterozygous for *CG4936^EY10172^*, which is indicative of suppression as *csw^lf^* is recessive lethal. Altogether, these data suggest that *CG4936* acts on *PTP-ER* transcription and that this action has bearing on RAS/MAPK dependent developmental processes.

Excepting *CG4936*, most of the other candidates positioned downstream of MEK (39) caused a decrease in MAPK protein levels (<−0.25 log_2_-fold and *p*<1×10^−5^) (Table S5). A few of these factors caused a significant and reproducible reduction in *mapk* transcript levels as well, without significantly impacting the levels of other RAS/MAPK pathway components (Figure S4A; Table S5). These factors include two EJC components (*eIF4AIII* and *mago*) as well as three other candidates: *Cdk12*, *Fip1*, and *CG1603*. Depletion of these latter candidates led to a significant drop in *mapk* transcript levels as measured by qPCR (Figure 4A; Table S6). However, these three factors could be distinguished from the EJC components by the fact that they did not cause a shift in the *mapk* RT-PCR profile (Figure S5), but only a decrease in the overall transcript abundance, suggesting that they do act at a different step of *mapk* expression. Finally, lethal alleles of *CG1603* and *Fip1* dominantly suppressed a *Ras^V12^* induced rough eye phenotype (Figures 6H and S8A). The *CG1603* allele also suppressed the extra wing vein phenotype caused by *Egfr^Elp^*, as did *Cdk12^KG05512^* (Figure S8E and S8J). However, none of the alleles had a readily observable impact on *csw^lf^* phenotypes (unpublished data); although *Fip1* did cause a slight enhancement of *csw^lf^* lethality (Figure S8I). Finally, consistent with our cell culture data, knockdown of *CG1603* in larval imaginal disc clones was found to cause a pronounced decrease in MAPK protein levels (Figure 7C).

*Cdk12* is the main RNA polymerase II C-terminal domain kinase. Phosphorylation of the C-terminal domain of RNAP II is required for transcription elongation, RNA processing, and splicing [76],[77]. Thus it is possible that *Cdk12* influences either of these steps in the case of *mapk* expression although it does not seem to induce AS. CDK12 has previously been shown to function in conjunction with Cyclin L in regulating AS [78]. Consistent with these data, the *Drosophila* CycL ortholog, *CG16903*, was also a hit in our screen that mapped to the MEK-MAPK interval (Table S3) and caused a shift in the *mapk* RT-PCR profile (Figure S5). However, *CG16903* did not significantly change *mapk* transcript levels measured by qPCR (Table S5).

*Fip1* is also involved in transcript processing; its best studied ortholog in yeast is part of the pre-mRNA cleavage and polyadenylation complex [79]. mRNA polyadenylation plays an important part in stabilizing spliceosome assembly on the 3′ most exon of the transcript, is an important pre-requisite for effective mRNA export, and also influences mRNA stability [80]–[82]. Thus, like *Cdk12*, *Fip1* may be regulating *mapk* expression by controlling transcript abundance or by influencing splicing efficiency.

*CG1603* encodes a protein of unknown function that contains MADF type zinc finger domains that are related to Myb DNA binding domains [83]. *CG1603* is poorly conserved in humans, bearing distant homology to ZNF664 and ZNF322. Interestingly, ZNF322 was found to act as a transcriptional co-activator of SRF and AP-1 in humans, which would position it downstream of MAPK signaling [84], thus representing a potential feedback mechanism.

The majority of the factors that were observed to lower MAPK levels were either spliceosome components or factors associated to the splicing machinery (Figure 5A; Table S5). Intriguingly, multiple lines of evidence suggested that these splicing components can also play a specific role in modulating RAS/MAPK signaling: (1) most splicing factors did not have any detectable impact on CNK, AKT, and RAS levels, even though these proteins are all derived from intron-containing genes (Figure S6), (2) depletion of most splicing factors by RNAi did not significantly modulate PGN (peptidoglycan) and RAC1^V12^-induced JNK activation (Figure 3A; Table S4), and (3) these splicing factors scored as hits in previous screens less often than *bona fide* RAS/MAPK pathway components (Table S4). Accordingly, the majority of these splicing factors were categorized in the high specificity group. Importantly, many of these had impacts on MAPK levels that were comparable to or greater in strength than those splicing factors of the lower specificity group. This finding suggests that the higher specificity score is not simply attributable to lower knockdown efficiency or a weaker impact on constitutive splicing.

Interestingly, one indication that the canonical splicing factors might be acting differently on MAPK signaling than the EJC came from our qPCR expression data. Namely, some high specificity splicing factors such as the *CG10754* (the counterpart of human SF3A2, a U2 small nuclear ribonucleic particle [snRNP] associated factor involved in branch point binding [85]), *CG3198* (the ortholog of LUC7L3, a predicted splicing factor [86]), *Prp19* (the central component of the PRP19 spliceosomal complex involved in C complex assembly [87]), as well as *CG4849* and *CG6686* (two predicted tri-snRNP components [88],[89]) all caused a reduction in MAPK protein levels comparable to the EJC factors *mago* and *eIF4AIII*. However, no reduction of *mapk* mRNA was observed by qPCR (Figure 4A; Table S5). Furthermore, another important difference between the EJC and the canonical splicing factors was that the depletion of many candidates of the latter group caused an increase in nuclear pre-mRNA retention as measured by fluorescence in situ hybridization using a poly-A probe (Figure S4B and S4C) [90]. This indicates—as might be expected—that most of these splicing factors also play a more general role in splicing.

**Figure 8:**
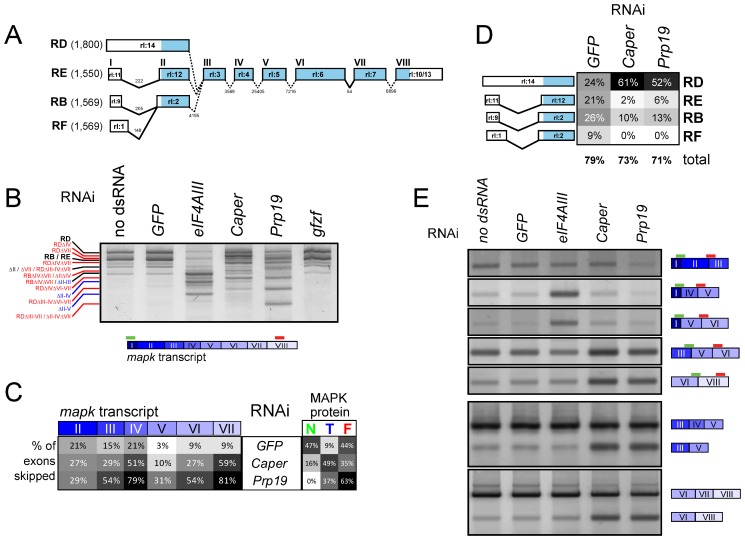
*Prp19* and *Caper* regulate *mapk* AS. (A) Schematic representation of the four annotated *mapk* splice isoforms observed in S2 cells. Exons are numbered from I to VIII based on the rl-RE transcript for simplicity in addition to the official Flybase exon names (e.g., “rl:14”). Introns lengths are also indicated. (B) An RT-PCR assay encompassing the entire *mapk* transcript (primers bind in exons I and VIII) for the four principle *mapk* isoforms is used to evaluate changes in the *mapk* transcript. In the untreated and *GFP* dsRNA controls, the two most abundant bands on the gel correspond to the RD isoform (topmost) and the RB/RE isoform (immediately below RD). Both *Caper* and *Prp19* knockdown are found to cause important shifts in the abundance and length of the *mapk* transcript (red labels), which differ from those produced by the depletion of the EJC component, *eIF4AIII* (blue labels). By default, all labels refer to the RB/RE isoform unless otherwise indicated. (C) Sequencing of the RT-PCR products from (B) reveals that the shorter products can be attributed to exon skipping events. In particular, exons IV and VII are the most frequently skipped following *Caper* and *Prp19* knockdown. This contrasts with *eIF4AIII* knockdown where we previously observed skipping of multiple consecutive exons [41]. The proportion of normal “N,” truncated “T,” and frameshifted “F” protein products is also indicated for the sequenced transcripts. (D) *Caper* and *Prp19* cause a shift from the RB/RE/RF forms towards the RD form, which is characterized by retention of the first intron. (E) Exon-exon junction spanning primers are used to detect specific exon skipping events (top panels). Skipping of exons II–III (second row) and exons II–IV (third row) is more abundant in *eIF4AIII* depleted cells. Exon IV (fourth row) and exon VII (fifth row) skipping is more prevalent following *Caper* and *Prp19* knockdown. Exon IV and VII skipping could also be detected using assays in which both primers lie within an exon region (bottom panels).

The key difference between the EJC and canonical splicing factors suggested by the qPCR and pre-mRNA export data was readily observable using the whole-transcript *mapk* RT-PCR assay; both groups caused an alteration in the *mapk* RT-PCR product, but the canonical splicing factors produced a clearly different pattern (Figure S5). Since most of the candidates in the canonical splicing factors group produced similar shifts in the *mapk* RT-PCR profile, we selected two representative factors for more detailed follow-up experiments. The first, *Prp19*, was selected because it is a core spliceosome component that caused a strong reduction in MAPK levels and had a clear impact on the *mapk* RT-PCR profile (Figures 5A, 5B, and 8B). The second, *Caper*, was selected because it is a serine rich (SR) AS factor [91] that did not perturb global pre-mRNA export, and it caused a weaker reduction in MAPK protein levels and a less severe change in the *mapk* RT-PCR profile (Figures 5A, 5B, 8B, and S5). Consistent with the protein expression data, while both factors clearly altered the *mapk* RT-PCR products, similar RT-PCR assays targeting *Ras85D*, *raf*, *mek*, *cnk*, or *ksr* did not display any obvious change (unpublished data). Also, Western blot experiments showed a clear drop in MAPK protein levels with no effect on RAS, RAF, MEK, or AKT (Figure 5B); this suggests that splicing of *mapk*, and not of other pathway components, is affected.

Also lending strength to the idea that splicing factors may have a specific role in the RAS/MAPK context was the fact that three *Prp19* alleles and one *Prp8* allele, were isolated by our group in an independent genetic screen for modifiers of a dominant-negative form of CNK (Figure S8B and CB, ML, MS, and MT, unpublished data). In addition to this finding, *Prp8* has been previously found to enhance the small wing phenotype induced by expression of the *Egfr* inhibitor *aos* [92]. Consistent with this result, the *Prp19* and *Prp8* alleles dominantly suppressed the RAS^V12^ rough eye phenotype, as did an allele of *Caper* (Figures 6I, 6J, and S8A). The *Prp19* alleles also dramatically enhanced *csw^lf^* lethality and suppressed the *Egfr^Elp^* wing phenotype (Figure S8E, S8I, and S8J). Moreover, in *mapk/rl^1^* homozygous flies, *Prp19* and *Caper* alleles enhanced the severity of wing vein deletions and rough eye phenotypes (Figures 6K–6R, S8G, and S8H). Importantly, although flies carrying one copy of both *Caper^f07714^* and *Prp19^CE162^* were perfectly viable, this allelic combination was entirely lethal in a *mapk/rl^1^* homozygous background (Figure S8G). This result constitutes another indication that these splicing factors are acting in concert on *mapk* expression. Finally, splicing of *mapk* was found to be altered in *Prp19^CE162^* flies also homozygous for *rl^1^* (Figure S10A and S10B). The *rl^1^* mutant alone reduces *mapk* transcript levels without altering the RT-PCR splicing profile [93; and unpublished data].

Complementing the genetic interaction experiments, expression of *Prp19* RNAi reduced the *Ras^V12^*-induced proliferation of larval hemocytes (Figure 7A). In addition, clonal tissue expressing *Prp19* RNAi in wing imaginal discs consistently caused a reduction in MAPK levels (Figure 7D), although the reduction was not as pronounced as that of *mapk* RNAi. Clonal regions sometimes showed signs of apoptosis (in one of the two RNAi constructs tested and in wing discs in particular) suggesting that these tissues may be more sensitive to knockdown of *Prp19* than S2 cells. Finally, splicing of *mapk* was found to be altered in wing disc segments where *Caper* had been knocked down (Figure S10C).

Altogether, these experiments suggest that these transcription and splicing factors are important in regulating MAPK levels, and thus are important for MAPK signaling. Furthermore, our genetic interaction data suggests that they can act in a number of different *in vivo* contexts. In particular, the *Prp19* alleles had an impact in all our RAS/MAPK genetic interaction experiments and displayed some of the strongest phenotype modifications. Thus, of the different groups of candidates, it is quite possible that splicing factors are relevant to the broadest range of RAS/MAPK regulatory and developmental contexts.

### Analysis of mapk AS Induced by Caper and Prp19 Depletion

*Prp19* and *Caper* RNAi display similar *mapk* RT-PCR profiles, but *Caper* produces a more subtle shift in product size with less lower size bands observable (Figure 8B). To verify that these changes in the RT-PCR profile of *mapk* were due to altered splicing, we cloned and sequenced the RT-PCR products. We found that the lower size *mapk* transcripts produced by both *Caper* and *Prp19* RNAi were generated by a series of exon skipping events and, to a lesser extent, by retention of the 5′ most intron of the RB/RE isoforms (Figure 8A–8D; Table S7). *Prp19* and *Caper* RNAi produced many single exon skipping events with exons IV and VII being the most frequently skipped. These AS events differed from those we had previously observed in EJC depleted samples, where skipping of multiple consecutive exons was observed immediately to the 3′ end of exon I [41]. Interestingly, exon skipping events associated with the EJC mostly resulted in frameshifting due to the loss of the start site in exon II and/or skipping of exon III. On the other hand, the skipping of exons IV and VII associated with *Prp19* and *Caper* produce an in-frame deletion potentially giving rise to a truncated protein product. The fact that we did not observe any smaller size products may be due to the epitope being removed or to the smaller products being unstable and degraded (MAPK is mostly composed of a kinase domain and it is likely that these deletions would disrupt proper folding).

In order to confirm that depletion of *Prp19* and *Caper* produced AS changes in *mapk* that were different from those produced by EJC depletion, we designed RT-PCR assays aimed at detecting specific AS events. Using primer pairs in which the 5′ primer overlapped the exon junction between exons I and III or exons I and IV, we were able to detect an enrichment in exon II–III and II–IV skipping in *eIF4AIII* depleted samples, consistent with our previous results. However, these exon skipping events were not enriched in *Caper* and *Prp19* RNAi samples. Conversely, using the same strategy, increased skipping of exons IV and VII was observed for *Caper* and *Prp19*, but not for *eIF4AIII* (Figure 8E). Also, using primers targeting the exons on either side of the skipped exons, we were able to detect both the canonical and the alternative transcripts produced by either *Prp19* or *Caper* depletion in both S2 cells and wing disc tissue (Figures 8E and S10C). In sum, multiple lines of evidence indicate that *Prp19* and *Caper* cause specific changes in *mapk* splicing that differ from those associated to the EJC. This finding suggests that at least two different types of regulation act on *mapk* AS. By extension, it is likely that the other splicing factors identified in our screen can be grouped with *Caper* and *Prp19*—and not the EJC—since they produced *mapk* RT-PCR profiles similar to these factors.

## Discussion

**Figure 9:**
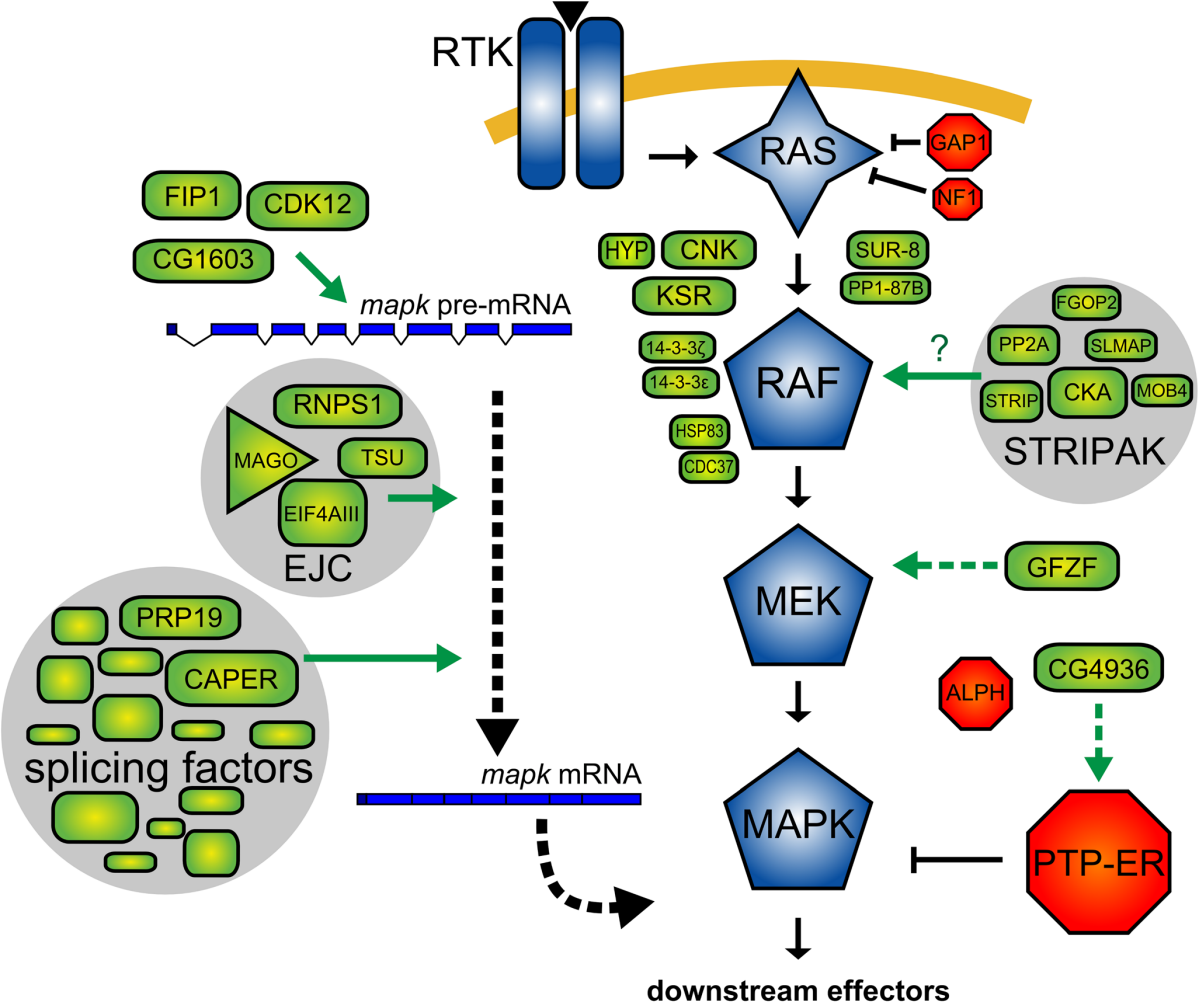
Regulatory input at the level of MEK, PTP-ER, and MAPK expression adds another layer to the network of factors that control RAS/MAPK signaling. Schematic model of proteins associated with RAS/MAPK signal transmission discussed in this work. Components used in secondary screens (GAP1, NF1) are also depicted. *Sur-8*, *PP1-87B*, and the five STRIPAK complex components were positioned between RAS and RAF in our epistasis assays. Their position would be consistent with a role in the RAF activation process. As it has been previously shown in mammalian models, SUR-8 and PP1 may be acting on RAF activation by dephosphorylating the N-terminal 14-3-3 binding site. Because PP2A is also known to dephosphorylate this site and because STRIPAK has been characterized as a PP2A-associated complex, STRIPAK may be involved in facilitating PP2A binding to RAF. GFZF was positioned at the level of MEK and was found to impact MEK expression, presumably by regulating *mek* transcription. CG4936 was found to impact expression of the MAPK phosphatase, PTP-ER, and also probably acts at the level of transcriptional regulation. CG1603, FIP1, and CDK12 were found to act on MAPK expression, most likely acting as transcriptional regulators (CG1603) or involved in transcript maturation/processing (FIP1 and CDK12). Finally, components of the spliceosome, splicing factors and the EJC were found to modulate MAPK expression by altering the splicing of the *mapk* transcript. The particular sensitivity of MAPK to disruption of these spliceosome components may be due to their involvement in recruiting specific mRNA processing factors such as the EJC. Alternatively, the reason why *mapk* displays an increased requirement for this set of spliceosome components may be due to a feature in *mapk*'s gene structure. For example, intron length is correlated with sensitivity of transcripts to EJC depletion [41].

In this report, we presented the results of an RNAi screen for factors influencing signaling between RAS and MAPK in *Drosophila*. Most previously known pathway components have been identified in our screen, including a few that had not yet been found in flies (e.g., *Sur-8*, *Pp1-87B*, *Hmgcr*, and *Fnta*). On the basis of analyses of our secondary screen results and publicly available data, we assessed the specificity of our candidates, grouped them into protein complexes, and positioned their effect relative to the core RAS/MAPK pathway components (Figure 2). We also evaluated the impact of our candidates on the expression of core components of the RAS/MAPK pathway. From these data, we identified distinct groups of factors with different roles in modulating RAS/MAPK signaling. Furthermore, we show that the largest group of candidates—which is composed of splicing factors—acts specifically on *mapk* expression. This discovery is surprising since most of the previously described regulators of MAPK signaling act at the level of RAF activation. Thus, our results uncover an unappreciated point of control governing *Drosophila* RAS/MAPK signaling that takes place through the control of *mapk* expression (Figure 9). Moreover, the identification of two other factors (*gfzf* and *CG4936*) that act to control *mek* and *PTP-ER* expression adds another layer to the gene expression control network surrounding the RAS/MAPK pathway.

When comparing our results to those of three previous RNAi screens examining insulin receptor (InR) and EGFR induced MAPK signaling in *Drosophila* cells in culture [37],[62], we found that our validated hit set had a relatively limited overlap with those studies; 44 of our 78 positive regulators were present in their list of 986 positive regulators and 18 of our 28 negative regulators were also present in their group of 1266 negative regulators (Figure S11A and S11B). 331 hits reported in the InR screen were tested for their ability to modulate RAS^V12^ in an assay similar to the one used in our screen. However, very few of the candidates found to modulate RAS^V12^ signaling in their secondary screen were also present in our validated hit set; 14 of our 106 validated genes were found to alter RAS^V12^ induced pMAPK beyond 5% of controls in their study (Figure S11C and S11D). Furthermore, nine of these 14 genes were *bona fide* pathway components. This limited overlap between our studies can be explained by two things: First, their S2R+ InR assay involved the use of an exogenous source of YFP-tagged MAPK where the YFP signal was used to normalize pMAPK signal. This strategy makes detection of factors that modify endogenous MAPK expression impractical (a large proportion of our candidates are involved in exactly this type of regulation). Second, when selecting which hits to follow up in secondary screening, the authors elected to exclude certain genes linked to large molecular complexes, most likely excluding many of the splicing factors that were retained in our study.

### Control of mapk Expression

With the exception of the well described regulation of RAS by *let-7* family miRNAs [22], surprisingly little is known on the regulation of MAPK module component expression. It was therefore surprising to find that our largest group of hits specifically decreased MAPK levels. Yet, control of MAPK expression is not unprecedented. For example, in yeast, both the FUS3 and HOG1 MAPKs are transcriptional targets of their respective pathways [94]. Pumilio mRNA binding proteins are also known to reduce MAPK activity by lowering the expression of the *C. elegans* MAPK, Mpk-1, as well as ERK2/MAPK1 and p38α/MAPK14 in human embryonic stem (ES) cells, through the binding of specific sites on the 3′ UTR of their respective transcripts [95]. Finally, LARP-1 RNA-binding proteins have been found to control the abundance of the transcripts of Mpk-1 and other pathway members in the *C. elegans* germ line [96].

In this study, we found that multiple splicing factors act downstream of MEK to specifically control MAPK levels. While some of these factors were associated with the regulation of AS, most were components of the spliceosome or factors that co-purify with the spliceosome. Interestingly, many lines of evidence suggest that AS can be modified by spliceosomal factors [97]–[106]. Global analyses of splicing events in yeast using a series of temperature sensitive alleles and deletions of splicing factors, found that spliceosome components differed in terms of which splicing events they altered [98],[99],[105]. Among these, the yeast homologs of *Prp19*, *Prp8*, *U4-U6-60K*, *l(3)72Ab*, *Prp6*, *SmD2*, *SF3a120*, *CG4849*, and *CG10333*—factors included in our set of hits—were found to differentially modulate the splicing of specific sets of transcripts [98]. Moreover, studies conducted in *Drosophila* using an RNAi-based strategy have demonstrated that knocking down many so-called “core” spliceosome components caused specific changes in specific AS contexts [100],[101]. In particular, some of the “core” components identified in our screen have been shown to have selective effects. For example, knocking down *Prp6*, *l(3)72Ab*, *crn*, *Caper*, *SF3a120*, and *CG10418*, was found to differentially influence AS of *Dscam*, *para*, *TAF1*, and/or *dAdar* [100],[101]. In mammals, SmB was also recently shown to be involved in regulating inclusion of alternative exons [103]. Finally, our observation that a *Prp19* knockdown had specific effects on MAPK protein levels and the identification of *Prp19* (as well as *Prp8*) in a separate CNK-based genetic screen supports the notion that these splicing factors are important for *mapk* expression *in vivo*. Thus, the fact that we identified specific spliceosome components in our screen and not others may reflect the importance of these particular components in regulating *mapk* splicing.

The specific elements of the *mapk* gene structure that dictate requirement of particular splicing factors have yet to be determined. Likewise, many—if not most—other genes may have structures that preclude AS regulation by components of the spliceosome. Still, AS processes in at least four other *Drosophila* genes seem to involve some of the same components of the splicing machinery that we have linked to *mapk*, indicating that a common characteristic may dictate the involvement of these spliceosome components in AS. On the other hand, the fact that some of the spliceosome factors are involved in specific AS contexts and not others, suggests that key differences exist. This observation is important as it implies that specific AS events can be modulated by controlling the activity of these spliceosomal factors. In support of this idea, one of the previously mentioned studies indicates that the control of *TAF1* AS by a series of splicing factors—which include *Caper* and other spliceosome components—is downstream of a Camptothecin-induced ATR pathway [101]. Another example is the regulation of CD44 AS by RAS/MAPK-induced phosphorylation of Sam68, which has been found to function by regulating the activity of the U2AF65 spliceosome component [107]. In addition to Sam68, other splicing factors and spliceosome components—including some that were identified in our screen—were recently found to be the targets of ERK phosphorylation [108]–[110]. Finally, a general splicing repressor, SRp38, has also been shown to function as a sequence specific splicing activator upon phosphorylation in response to cellular stress [111].

One explanation for our results, as well as some of the previous observations, would be that some of the spliceosomal factors in our set may be interchangeable or function as non-essential co-regulators. Thus, removing these components would not greatly disrupt general spliceosome function, but rather lead to an altered spliceosome activity that selectively impacts sensitive AS contexts. An example of this is the interplay between PUF60 and U2AF65, which both function in 3′ splice site recognition; the two factors can either work cooperatively or independently, producing different splicing outcomes on the basis of the presence or absence of either protein [112]. A further example is the stress-induced relocalization of certain spliceosome and splicing factors that leads to changes in spliceosome configuration and AS [113]. Cellular stress has also been associated with the production of “non-productive” AS variants or silent messengers (transcripts that are either degraded by the quality control machinery or that do not encode functional proteins) [114],[115]. In fact, the AS we observed in *mapk* is reminiscent of the AS observed for the E3 ligase MDM2 following camptothecin-induced genotoxic stress [116]. In this study, the authors found that stress induced a number of non-productive MDM2 transcript isoforms that resulted in lower MDM2 protein levels and a stabilization of the MDM2 target, p53. It will be important to determine whether stress—or another signal—acts to control MAPK protein levels by inducing the AS we observe in our experiments.

The discovery that MAPK expression is specifically modulated by mRNA processing factors raises multiple questions. Not only will it be vital to define the upstream signals that dictate this activity but it will also be important to assess which other genes are similarly regulated and to identify the common characteristic that renders them sensitive to this type of regulation. Also, the time frame within which these changes occur will have implications as to the role that this type of regulation can play in MAPK signaling. Typically, RAS/MAPK signal modulation has been observed to occur through either rapid post-translational mechanisms or through slightly slower mechanisms involving control of protein stability and transcriptional control. However, the reduction in protein levels we observed as a consequence of AS are only apparent over a period of days, probably because the MAPK protein is relatively stable. This finding indicates that this regulation will not be relevant over the shorter timeframes of previously characterized regulatory events and also implies that a prolonged stimulus will be necessary to produce the effects we observe. Therefore, control of *mapk* splicing may be more important in the context of certain tissues and organs, in development or in disease. Interestingly, the abundance of core spliceosome components has been shown to be regulated and vary both temporally and across different tissues [117]–[121]. What is more, disruption in core spliceosome components has also been found to cause changes in AS in diseases such as spinal muscular atrophy and retinis pigmentosa [122]. Mutations in splicing factors have also been found to occur in a large proportion of myelodysplasia patients [104],[123] as well as in melanoma [106]. Another study has shown that, in glioblastomas and astrocytomas, splicing factors controlled by c-Myc play a role in controlling the expression of pyruvate kinase, a factor that is important for aerobic glycolysis [124]. It will be interesting to explore whether, in contexts such as these, the altered activity of splicing factors may regulate MAPK levels with important functional consequences for either normal or diseased cellular function.

## Methods

### Genome-Wide RNAi Screen

*pMet-Ras^V12^* S2 cells diluted in Schneider medium (to a concentration of 1×10^6^ cells/ml) were distributed in 96 well clear plates (Corning) containing 5 µl dsRNA aliquots at a concentration of ∼200 ng/µl. Plates were placed in plastic containers to reduce evaporation and incubated at 27°C for four days. *Ras^V12^* expression was induced by adding 0.7 mM CuSO_4_ to medium 24 h prior to fixation. Cells were resuspended and transferred to concanavalin A coated plates and allowed to settle and adhere for 1 h. Cells were then fixed in 4% paraformaldehyde/PBS, washed, and blocked in 0.2% Triton X-100/0.2% BSA/PBS (PBT/BSA) and incubated overnight with an anti-pMAPK antibody (1/2,000; Sigma number M8159), washed in PBT/BSA, and revealed using an anti-mouse Alexa Fluor 555-conjugated secondary antibody (1/1,000; Invitrogen number A-21424). DAPI (0.04 µg/ml) was used to stain nuclei. Mowiol (9.6% PVA, Fluka) was added to wells prior to imaging. An automated fluorescence microscopy system (Zeiss Axiovert) was employed for plate imaging. Autofocus, image acquisition, and analysis were conducted using MetaMorph (Molecular Devices) software. The cell-scoring application in MetaMorph was used for cell segmentation and quantification of fluorescent signal.

### Epistasis

Candidates were assigned to one of three possible epistasis intervals (RAS-RAF, RAF-MEK, or MEK-MAPK) on the basis of the data from the following secondary screens: (1) RAS^V12^, (2) RAF^ED^, (3) RAF^CT^, (4) RAF^EDCT^, (5) MEK^EE^, (6) MEK^EE^+PTP-ER dsRNA. The correlations between normalized secondary screen log transformed values and three predetermined epistasis profiles were calculated using a modified uncentered Pearson's correlation:
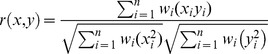
Where *r* is the correlation value [−1,1], *x* is the secondary screen value (for screens number 1, 2, 3, 4, 5, and 6), *y* is the predetermined profile value, and ***w*** is the weight applied to a given screening experiment (where *w* = [3 1 1 1 1 2]; see Text S1). The following predetermined epistasis profiles ***y*** were used:

RAS−RAF = [1 0 0 0 0 0]

RAF−MEK = [1 1 1 1 0 0]

MEK−MAPK = [1 1 1 1 1 1]

A negative *r* indicates reverse correlation and is observed for positive pathway regulators.

### Fly Genetics and Microscopy

Fly husbandry was conducted according to standard procedures. All crosses were performed at 25°C. The *sev-Ras^V12^* line has been described previously [45]. *Egfr^Elp^* was described in [125]. The *Cka* alleles [55] were kindly provided by S. Hou. The *csw^lf^* [126] flies were originally obtained from L. Perkins. The *mapk^E1171^* allele was identified in a genetic screen as a dominant suppressor of a dominant negative form of KSR [127]. The *Prp19^CE162^*, *Prp19^CE40^*, *Prp19^TE1036^*, and *Prp8^CE309^* alleles were recovered in a genetic screen for modifiers of a dominant negative form of CNK (CB, ML, MS, and MT, unpublished data).

RNAi fly lines were obtained from the VDRC [128]. All other fly lines described herein were obtained from the Bloomington stock center.

Adult fly eyes were imaged using a stereomicroscope (Leica MZ FL III) and CombineZP, a freely available software package (http://www.hadleyweb.pwp.blueyonder.co.uk/CZP/News.htm), was used for focus stacking. Adobe Lightroom and GNU Image Manipulation Program (GIMP) were used for image processing. Wings were mounted in Permount (Fisher) on glass slides and imaged using a Nanozoomer (Hamamatsu).

RNAi clones were generated using a line carrying a heat shock inducible flip-out actin promoter driving the expression of GAL4 and GFP in clonal tissues (*hs-flp;; Act5C>CD2>Gal4, UAS-GFP*). L1 larvae were heat shocked for 15 minutes at 37°C and later collected for dissection upon reaching late L3 (wandering) stage. Third instar eye-antennal and wing discs were dissected in Schneider medium, fixed, and stained with DAPI and an anti-MAPK antibody (1/1,000, Cell Signaling number 4695) following the same procedure described above for S2 cells.

### *Drosophila* Gene Nomenclature

The following *Drosophila* genes are named on the basis of their human counterparts: *Fnta* (farnesyl transferase alpha; *CG2976*), *Fgop2* (fibroblast growth factor receptor 1 oncogene partner 2; *CG10158*), *Slmap* (sarcolemma associated protein; *CG17494*), *Strip* (Striatin interacting protein; *CG11526*). *Ras85D* (refers to the *Drosophila* gene encoding RAS), *ras* (gene encoding an IMP dehydrogenase) is referred to by its full name, “raspberry,” to avoid confusion. Following the nomenclature recommended by Flybase, gene symbols are in lower-cased italics and protein symbols are in upper-case without italics. *hyp* (hyphen; designates the *ave*/*hyp* gene).

## Supporting Information

Figure S1 **Primary screen and screening strategy.** (A) Primary screen hits are submitted to validation screening steps to eliminate false positives. Remaining candidates were submitted to secondary screens to assess the position of the regulatory input relative to known pathway components (epistasis) and specificity to the RAS/MAPK signaling context. In addition to this, candidates were screened for their impact on core RAS/MAPK component expression, both at the transcript (qPCR and RT-PCR) and protein level (quantitative immunofluorescence and Western blot). (B) The robustness of the primary screen assay was evaluated by monitoring changes in RAS^V12^-induced pathway activity following knockdown of *mek* and *PTP-ER*. The levels of pMAPK are then measured by quantitative microscopy. Results shown for each dsRNA are the mean of 43 sample wells in three separately prepared plates. The calculated Z′-factor was 0.643 for *mek* depletion and 0.175 for *PTP-ER* depletion. (C) Distribution of primary screen probe data organized in plate screening order (grey data points). *GFP* dsRNA (blue) was used as a negative control and reference to normalize screening plate data. *mek* and *PTP-ER* dsRNAs (green and red, respectively) were used as positive controls to verify dsRNA knockdown efficiency.
(TIF)

Figure S2 **Promoter validation screens.** (A–B) *pMet* promoter validation screen assays and control experiments. (A) *Ras^V12^* expression in *pMet-HA-Ras^V12^* stably transfected S2 cells is monitored by quantitative immunofluorescence through the use of anti-HA antibody. Values shown are the average HA signals of duplicate samples normalized to CuSO_4_ induced controls with no dsRNA treatment. (B) GFP expression in *pMet-GFP* stably transfected S2 cells is monitored by quantitative immunofluorescence. Values shown are the average GFP signals of duplicate samples normalized to CuS0_4_ induced controls with no dsRNA treatment. (C) Variation of pMAPK, GFP, and HA signal in response to increasing amounts of *MTF-1* dsRNA. Values shown are average of triplicate samples normalized to CuSO_4_ induced controls with no dsRNA treatment. (D–E) Promoter validation screen results (*x* axis) plotted against pMAPK primary screen values (*y* axis). The cutoff (dashed line) to identify false positive candidates with effects on promoter activity (data points in red areas) is a function of the pMAPK signal observed in the primary screen (see Text S1). MAPK regulators (red), STRIPAK (blue), EJC (green) as well as *Sur-8*, *Pp1-87B*, *Fnta*, and *Prp19* (black) are shown. Factors known to be involved in *pMet*-driven expression, such as *MTF-1*, TBP-associated factors (Tafs), and *Ctr1B* are also shown (grey). (D) *pMet-HA-Ras^V12^* validation screen results. The HA signal from the validation screen (*x* axis) plotted against the pMAPK signal from the primary screen (*y* axis). The HA and pMAPK values shown are normalized to plate-specific *GFP* dsRNA controls. (E) *pMet-GFP* validation screen results. The GFP signal from the validation screen (*x* axis) plotted against the pMAPK signal from the primary screen (*y* axis). The GFP values shown are normalized to plate-specific *mek* dsRNA controls. The pMAPK values shown are normalized to plate-specific *GFP* dsRNA controls.
(TIF)

Figure S3 **Secondary screens.** (A) MAPK and JNK pathway models depicting the secondary screen assays used in this study. See Text S1 for screen descriptions. (B–N) Secondary screen control assays. Values shown are duplicate sample averages normalized to *GFP* dsRNA treated controls unless otherwise indicated. (B–K) are MAPK activation assays with a pMAPK readout. (L–N) are JNK pathway assays with a pJNK readout. (B) RAF-based MAPK activation: Values shown are for *pMet-raf^ED^*, *pMet-raf^CT^*, and *pMet-raf^EDCT^* stably transfected cell lines treated with the indicated dsRNAs and induced with CuSO_4_ for 24 h. (C) MEK-based MAPK activation: dsRNA treated *pMet-mek^EE^* stably transfected cells were induced with CuSO_4_ for 24 h. (D) RAS GAP RNAi-based MAPK activation: S2 cells were treated with dsRNAs targeting the indicated RAS GAPs or predicted RAS GAPs. Values are normalized to untreated control samples. Combined knockdown of *Gap1* and *Nf1* produced the highest pMAPK activation and was used in the secondary screen assay. (E and F) Insulin-based MAPK activation. (E) Insulin induction time course: The values shown are baseline normalized signal averages from single well samples of insulin treated S2 cells induced for the indicated times. (F) The signals shown are the average from dsRNA-treated single well samples of insulin induced S2 cells induced for 5′. (G and H) SEV^S11^-based MAPK activation. (G) Heat shock induction time course of *pHS-sev^S11^* stably transfected S2 cells. Samples are induced for 30 minutes at 37°C and incubated at 27°C for the indicated times before sample preparation. Values shown are normalized to a non-induced control. (H) dsRNA-treated *pHS-sev^S11^* cells were induced with a 30′ minutes heat shock at 37°C followed by a 2.5 h incubation at 27°C. (I–K) EGFR-based MAPK activation assays. (I) Induction time course was performed on *pMet-Egfr* stably transfected cells induced with 1∶2 supernatant from *pMet-Spi* cells. Values are normalized to non-induced controls. (J) Calibration of Spitz concentration: *pMet-Egfr* cells were induced for 5′ with the indicated dilution of Spitz supernatant and normalized to 1∶1 Spitz induced controls. (K) dsRNA-treated *pMet-Egfr* cells were induced for 5′ with the indicated concentrations of Spitz supernatant. Values shown are normalized to samples not treated with dsRNA. (L and M) RAC1^V12^-based JNK activation time courses. (L) Induction time course: *pMet-Rac1^V12^* stably transfected cells were induced with CuSO_4_ for the indicated times. Values shown are from triplicate samples of two experiments. (M) *pMet-Rac1^V12^* cells were treated with *hep* dsRNA and induced with CuSO_4_ for the indicated times. Values shown are from single well samples normalized to samples not treated with dsRNA. (N) Peptidoglycan (PGN) based JNK activation time course: S2 cells were induced with 50 µg/ml LPS for the indicated times. The values shown are averages of four samples from two separate experiments.
(TIF)

Figure S4 **qPCR screen.** (A) Unsupervised hierarchical clustering of qPCR screen results. SYBR green qPCR assays were used to assess the levels of RAS/MAPK pathway transcripts (top labels) following depletion of the indicated *Ras^V12^* screen candidates (labels, left). The transcript levels are presented as log_2_ transformed ratios of *GFP* dsRNA treated controls. RAS/MAPK control dsRNAs are indicated by asterisks. The *gfzf* dsRNA has the closest profile to the *mek* control dsRNA, with both reagents causing a reduction in *mek* levels. *PTP-ER* and *CG4936* also have very similar profiles and cause a reduction in *PTP-ER* transcript levels. As we previously reported, the EJC components (blue) *mago* and *eIF4AIII* cause an observable decrease in *mapk* transcript levels. However, the knockdown of *tsu* and *RnpS1*, which have a weaker impact on pMAPK and MAPK levels, does not cause a readily observable change in *mapk*. Only *CG1603* and *Cdk12* had similar profiles to the two stronger EJC components, causing a decrease in *mapk* transcript levels. The *Fip1* dsRNA results in the qPCR screen were not valid, but *Fip1* dsRNA was re-tested in the confirmation qPCR experiment presented in Figure 2 and found to have a similar profile to the two other factors. Most of the splicing factors—other than the two EJC components—were not found to cause changes in the levels of *mapk* or any of the other RAS/MAPK transcripts. (B) Total mRNA FISH performed using an oligo-dT Cy3-labeled probe. The proportion of cells displaying nuclear retention was evaluated by performing segmentation and scoring the overlap of the FISH signal with a DAPI nuclear stain. dsRNA targeting *sbr*, the homolog of the mammalian nuclear RNA export factor 1 (NXF1), and *CG2063*, a factor previously linked to mRNA export, both caused an increase in nuclear retention. In comparison *mago* dsRNA did not visibly alter total mRNA retention. (C) Total mRNA export screen results. A subset of 44 candidates were tested to evaluate their impact on total mRNA export: these included all factors with functions linked to mRNA splicing (red) as well as other factors found to alter MAPK protein or *mapk* mRNA levels. A nuclear retention index was calculated by normalizing the rate of nuclear retention to the average results of *GFP* dsRNA treated controls (green). *sbr* dsRNA was used as a positive control (red arrowhead) for nuclear retention. In addition to *sbr*, five candidates in our set (orange circles) were also identified in a mRNA nuclear export RNAi screen [90].
(TIF)

Figure S5 **RT-PCR screen.** An RT-PCR assay encompassing the entire *mapk* RB, RD, RE, and RF transcripts was used to evaluate the impact on the distribution of *mapk* transcript isoforms. The same subset of 44 hits selected for the mRNA export screen were also tested here. A majority of factors tested caused a change in the *mapk* RT-PCR profile when compared to *GFP* dsRNA treated or untreated controls. The altered profiles were grouped into two categories (A and B) on the basis of similarities in the sizes of the bands observed. Most splicing factors in the set produced an “A” type profile while EJC factors produced a different “B” type profile. Other candidates causing no obvious change in product size, but causing a reduction in band intensity are labeled “R.” Those causing no observable change are labeled “N.” The labels on the right of the bottom panel refer to *mapk* transcript isoforms that correspond to the bands visible on the gel. Candidates featured in the manuscript are labeled with a green star.
(TIF)

Figure S6 **Immunoblot screen.** (A–M) Western blot analyses of S2 cells treated with the indicated dsRNAs. Endogenous levels of CNK, AKT, MAPK, and RAS were monitored using specific antibodies. A negative (*GFP* dsRNA) and two positive (*mago* and *eiF4AIII dsRNAs*) controls were included in this experiment.
(TIF)

Figure S7 **Co-immunoprecipitation of Drosophila STRIPAK complex components.** S2 cells were transfected with expression plasmids carrying cDNAs encoding the fusion proteins indicated at the top of (A, B, and C). Cell lysates were immunoprecipitated with the indicated antibodies (bottom of upper panels in (A and B), and left of upper panels in (C)). The immunoprecipitates and equal amounts of cell lysates (normalized for total protein content) were fractionated by SDS-PAGE and immunoblotted with the antibodies indicated at the right (A–C). The tagged STRIPAK complex protein shown in each panel is indicated in parentheses. None of the STag fusion proteins or GFP-FGOP2 are present in α-HA or α-HSV immunoprecipitates when these fusion proteins are expressed alone, and GFP does not co-immunoprecipitate with HA-CKA, HSV-MOB4, HSV-STRIP, or HSV-SLMAP (unpublished data).
(TIF)

Figure S8 **Additional genetic interaction data.** (A) *Ras^V12^* genetic interaction data for additional alleles of *Cka*, *gfzf*, and *Prp19* as well as alleles of *Fip1* and *Prp8*. (B) Details of molecular lesions present in the three alleles of *Prp19* and the *Prp8^CE309^* allele identified in a *cnk* dominant negative genetic screen (CB, ML, MS, and MT, unpublished data) and used in our genetic validation experiments. A protein map of PRP19 and PRP8 showing the amino acid changes found in the *Prp19* and *Prp8* alleles is presented with “*” indicating residues conserved in humans. The mutation in *Prp8^CE309^*, in addition to causing an amino acid change, is located on the second residue of a 3′SS (T4088A) and may impact splicing of *Prp8*. (C) Additional *rl^1^* rough-eye phenotype genetic interactions for *CG4936*. Trans-heterozygous *CG4936^DG10305^*/*CG4936^EY10172^* suppresses the weak rough eye phenotype of *rl^1^* homozygotes. Eye size is also slightly restored. (H) Representative wings for *csw^lf^* hemizygous males scored in (K). The deletion of the distal end of the L2, L3, and/or L5 vein is a frequently observed phenotype in a *csw^lf^* background. The occurrence, number, and severity of this deletion are more pronounced in *Cka^2^* and *gfzf^CZ811^* heterozygous backgrounds. (E) Representative wing images for the *Egfr^Elp^* males scored in (J). The additional wing vein near the extremity of the L2 vein (arrow) is characteristic in *Egfr^Elp^* flies. Wings displaying a suppressed phenotype (reduced frequency and length of the extra vein material) are shown for *Cka^2^*, *CG1603^f04743^*, *Prp19^TE1036^*, and *mapk*/*rl^1^* (positive control) heterozygotes. (F) Image of a wild-type fly wing with labels indicating the location of the five wing veins (L1–L5) and of the anterior (acv) and posterior (pcv) cross-veins. (G) Proportion of *rl^1^*/*rl^1^* males to *rl^1^*/*CyO* males observed following a *rl^1^*/*CyO* X *rl^1^*/*CyO* cross. The wings of *rl^1^*/*rl^1^* flies have a “rolled” phenotype; they are slightly curved downwards along the anterior-posterior axis. *rl^1^*/*CyO* flies display a regular *CyO* phenotype; pronounced upward curve along a lateral axis. Both *rl^1^ Prp19^CE162^*/*CyO* and *Caper^f07714^ rl^1^*/*CyO* were crossed to *rl^1^*/*CyO* flies. The frequency of *rl^1^ Prp19^CE162^*/*rl^1^* and *Caper^f07714^ rl^1^*/*rl^1^* was lower than that of *rl^1^*/*rl^1^*. The *rl^1^ Prp19^CE162^*/*Caper^f07714^ rl^1^* combination was not viable at 25°C. Trans-heterozygous *CG4936* slightly enhanced the proportion of *rl^1^*/*rl^1^* flies recovered. (H) *rl^1^*/*rl^1^* flies display a deletion of the mid-section of the L4 wing vein that is not fully penetrant. Examples are Shown for wings with weak “+” (single small deletion) and strong “++” (large or multiple deletions) wing vein deletions. Wing deletions were scored for the indicated genotypes. The total amount of individuals scored is indicated below the allele labels. (I) Proportion of *csw^lf^* males to *csw^+^* males observed for the indicated genotypes with the total amount of individuals scored indicated below the allele labels. The trans-heterozygous allelic combination of *CG4936* behaved similarly to heterozygous *CG4936^EY10172^* in *csw^lf^* genetic interaction experiments (wing vein deletions and viability) and is not shown here. (J) Proportions of wings from *Egfr^Elp^* flies with the indicated extra wing vein material phenotype severity for the indicated alleles. Examples of strong and weak additional wing vein phenotypes are shown next to the graph. The total number of wings scored is indicated below the allele labels. The *Fip1* and *gfzf* alleles displayed only slight suppression of the wing vein phenotype and are not shown here. (K) Proportions of wings from *csw^lf^* males with the indicated deletion severity for the indicated alleles. Examples of strong (large or multiple small) and weak (single, small) wing vein deletions are shown. The total number of wings scored is indicated below the alleles. The *Caper* and *CG1603* alleles only displayed very weak genetic interaction with *csw^lf^* and are not shown here. The *Prp19* flies produced too few escapers for accurate scoring of the wing phenotypes.
(TIF)

Figure S9 ***Cka*** **is important for photoreceptor development.** (A) *Cka^2^* mutant clones in pupal eye discs dissected 42 h APF are marked by the absence of GFP (A). The differentiated R7 photoreceptor is marked by the overlapping Elav and Pros stainings (in purple, A′ and A″). In *Cka^2^* clones, ommatidia lacking the R7 photoreceptor are marked with a white arrow. We also noted some displaced R7 photoreceptors (R7s outside the focal plane are marked with white arrowheads), and rotated ommatidia (white asterisks) in the *Cka^2^* clones.
(TIF)

Figure S10 ***In vivo*** **evidence for alternative splicing of** ***mapk*** **.** (A) Schematic representation of the *mapk* splice isoforms from Figure 8. (B) The RT-PCR assay spanning the whole *mapk* transcript was used to detect splicing changes in adult flies. Samples were prepared from five adult flies of the indicated genotypes. Escapers homozygous for *rl^1^/rl^1^* and also carrying the *Prp19^CE162^* mutant displayed splicing changes compared to a CantonS wild type control strain. The products from this experiment were not verified by sequencing though they were similar to those observed in S2 cells upon *Prp19* knockdown (the lower sized band in the WT control is assumed to be due to skipping of exon II, based on our S2 cell data). *rl^1^* homozygous flies have a reduced amount of *mapk* though the size of the RT-PCR product is unchanged (not shown). (C) *mapk* splicing is altered in larval wing imaginal discs following *Caper* knockdown. A RNAi construct targeting *Caper* was expressed in the posterior segment of wing imaginal discs using an *engrailed*-GAL4 driver. Imaginal discs from 3rd instar larvae were microdissected to separate the posterior (control) and anterior (*Caper* knockdown) segments. The RT-PCR assay spanning exons III–V was used on extracts from both samples to evaluate inclusion of exon IV. All *Prp19* RNAi tested with the *Engrailed-Gal4* driver caused a high rate of lethality and escapers that could be recovered were of reduced size and were not found to contain wing discs.
(TIF)

Figure S11 **Comparison with previous RTK/MAPK RNAi screens.** (A and B) Overlap of validated positive (A) and negative (B) regulators of RAS^V12^ identified in this study with positive and negative regulators reported in three Drosophila RNAi Screening Center (DRSC) RTK/MAPK screens [37],[62]. The diagrams include all genes reported as Insulin and EGF modulators (both in S2R+ and Kc cells) with a Z-score ±1.5 (986 positive and 1,266 negative regulators in total). The large Venn diagrams display hits in the three DRSC screens overlapping with hits reported here. The total hits reported in the DRSC screens are displayed in the small diagrams. Reported gene identifiers were first updated to the current annotation before comparison was performed. (C and D) Overlap of validated positive (C) and negative (D) regulators of RAS^V12^ identified in this study with 331 validated hits from the DRSC S2R+ insulin screen that were tested in a RAS^V12^ secondary screen [62]. 123 hits from the DRSC screen were reported to modulate pMAPK signal by over ±5% and were considered as hits for the purpose of this comparison.
(TIF)

Table S1 **Primary and promoter validation screen data (MS Excel file).** Primary screen and validation screen data. The average pMAPK observed in the *pMet-Ras^V12^* primary screen (from the primary and confirmation steps) are listed for the 309 initial primary screen hits. The associated values and results for the first of two validation screening steps (elimination of candidates impacting the *pMet* expression system using *pMet-GFP* and *pMet-HA-Ras^V12^*) are also shown.
(XLSX)

Table S2 **GO term enrichment.** GO terms found to be enriched in our set of validated hits using the Flymine [129] GO enrichment function. *p*-Values are calculated using Benjamini and Hochberg multiple hypothesis test.
(DOCX)

Table S3 **Epistasis screen results and calculated Pearson correlations (MS Excel file).** Results from the secondary screens used in the epistasis analysis are shown (screens number 1–6). Values shown are log_10_ transformed normalized pMAPK signals. Calculated Pearson uncentered correlation (*r*) are shown for the three epistasis profiles (RAS−RAF = [1 0 0 0 0 0], RAF−MEK = [1 1 1 1 0 0], MEK−MAPK = [1 1 1 1 1 1]). The final epistasis interval is also shown as well as the confidence score for the epistasis result. Candidates that were not positioned are marked with “?” while those with ambiguous positioning (confidence score <0.2) results are marked with “(A).”
(XLSX)

Table S4 **Specificity score results (MS Excel file).** The data used to calculate the specificity score are presented in the following order: number of times this gene has been identified in previous *Drosophila* RNAi screens; result observed in the Western blot screen (NS, non-specific effect; MAPK or RAS, specific effect on this component; - no effect); and associated specificity score; specificity score derived from the total mRNA nuclear export screen; score associated with a decreased cell count; effect on *pMet* driven expression derived from the *pMet-GFP* screen; JNK specificity result derived from the two pJNK secondary assays; combined specificity score derived from all secondary screen results; final specificity score and specificity group (high, medium, low). Specificity score calculation is described in the Text S1.
(XLSX)

Table S5 **Summary of secondary screen results (MS Excel file).** List of validated screen hits and secondary screen results. Values are expressed as log_2_ transformed ratios normalized to *GFP* dsRNA negative controls. Also presented in this table is: the calculated epistasis interval, specificity score, functional group (based on GO annotation), and predicted homologs in *Homo sapiens* and *Saccharomyces cerevisiae*. *Bona fide* RAS/MAPK components are marked with “*.” Genes to which we have associated new gene symbols are marked with an “†.”
(XLSX)

Table S6 **Detailed qPCR results (MS Excel file).** Values for the confirmation experiment and *in vivo* qPCR presented in Figure 5A and 5B. mRNA levels are expressed as log_2_ ratios of negative control (*GFP* dsRNA treated S2 cells or no RNAi flies). The right panel shows the associated *p*-values (unpaired two-tailed Student's *t*-test). *AGO1* and *sprt* were not followed-up as the observed increase in *mek* mRNA levels in the initial qPCR secondary screen was not confirmed in this experiment. The dsRNA probes from our set targeting *mapk* and *ksr* overlap with the SYBR green qPCR probes invalidating those results. Both qPCR assays were validated with separate dsRNAs (unpublished data).
(XLSX)

Table S7 **Detailed sequencing data from the mapk RT-PCR experiment (MS Excel file).** Summary of sequencing data from cloned *mapk* RT-PCR products. 142 clones were successfully sequenced and aligned in order to investigate the nature of the splicing defects induced by *Caper* and *Prp19* knockdown. *mapk* exons are labeled according to the numbering scheme we used in this study (I–VIII) as well as Flybase exon IDs. “rl:8/10/13alt” refers to an alternate exon located downstream of exon VIII and has been described previously [130]. Presence or absence of exons in a given clone is indicated by “1” or “0.” Exons not annotated in Flybase are indicated in the individual clone entries (for example, rl:2-B in clone CaperKD_41 is a rl:2-like exon with an alternate splice acceptor site). All exon sequences (annotated and novel) are listed in the “mapk exons” tab.
(XLSX)

Table S8 **qPCR and RT-PCR primers (MS Excel file).** Sequence of RT-PCR and qPCR primers used in this study. dsRNA primer sequences are available at the IRIC *RNAi* database (http://www.bioinfo.iric.ca/iricrnai).
(XLSX)

Text S1 **Notes on the exon junction complex and supplemental methods.** Document containing additional notes on the exon junction complex as well as an extended description of the experimental methods used in this study.
(DOCX)

## Abbreviations

AS: alternative splicing
dsRNA: long double-stranded RNA
EJC: exon junction complex
GFP: green fluorescent protein
GO: gene ontology
pMAPK: phosphorylated MAPK
qPCR: quantitative PCR
RTK: receptor tyrosine kinase
RNAi: RNA interference
STRIPAK: striatin-interacting phosphatase and kinase complex
