# Supplementary figures and images for: A Functional Screen Reveals an Extensive Layer of Transcriptional and Splicing Control Underlying RAS/MAPK Signaling in Drosophila
Source: PLoS Biol. 2014 Mar 18;12(3):e1001809. doi: 10.1371/journal.pbio.1001809 (PMC3958334; doi:10.1371/journal.pbio.1001809)

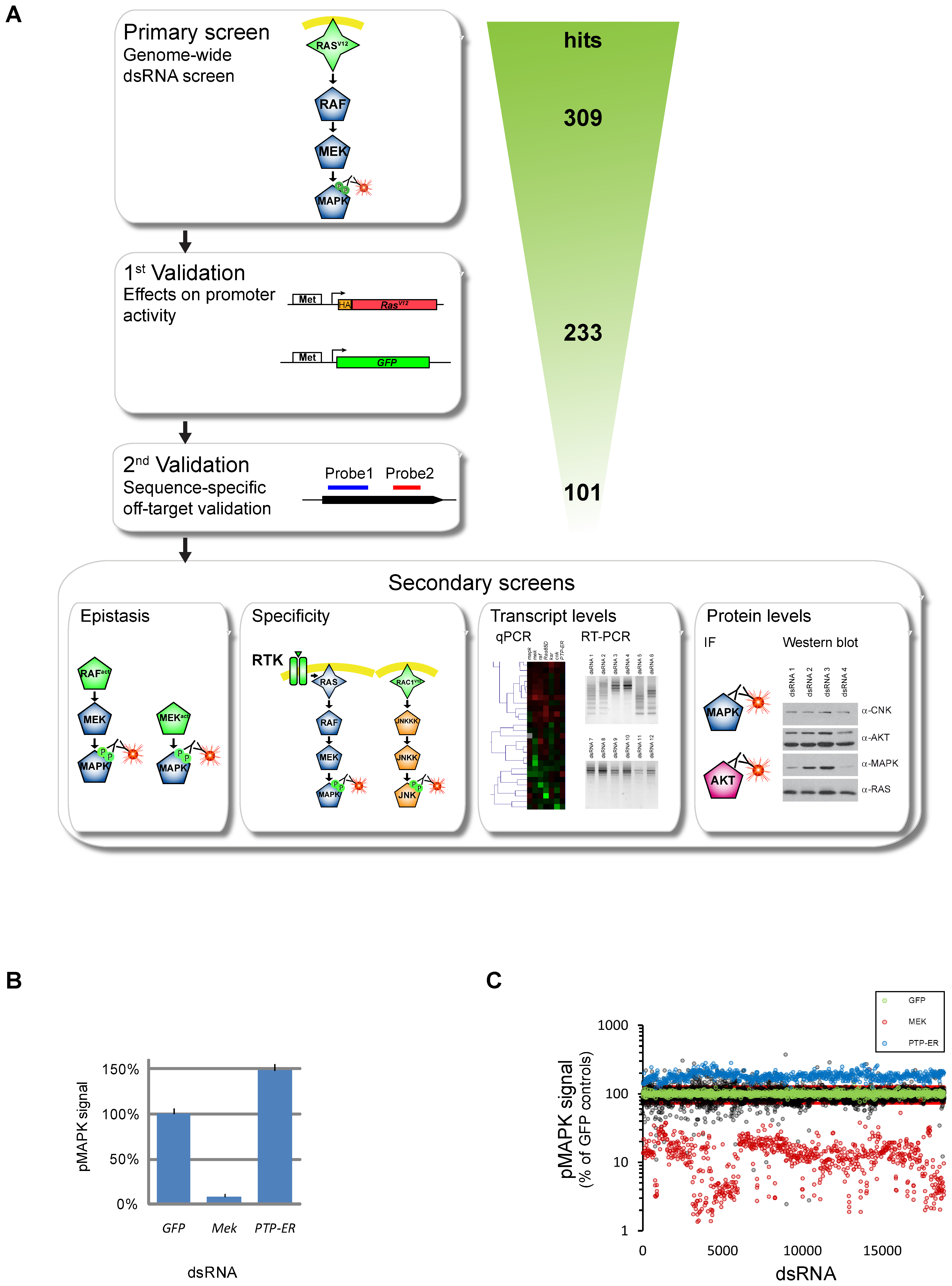

Supplement: Figure S1 — Primary screen and screening strategy. (A) Primary screen hits are submitted to validation screening steps to eliminate false positives. Remaining candidates were submitted to secondary screens to assess the position of the regulatory input relative to known pathway components (epistasis) and specificity to the RAS/MAPK signaling context. In addition to this, candidates were screened for their impact on core RAS/MAPK component expression, both at the transcript (qPCR and RT-PCR) and protein level (quantitative immunofluorescence and Western blot). (B) The robustness of the primary screen assay was evaluated by monitoring changes in RASV12-induced pathway activity following knockdown of mek and PTP-ER. The levels of pMAPK are then measured by quantitative microscopy. Results shown for each dsRNA are the mean of 43 sample wells in three separately prepared plates. The calculated Z′-factor was 0.643 for mek depletion and 0.175 for PTP-ER depletion. (C) Distribution of primary screen probe data organized in plate screening order (grey data points). GFP dsRNA (blue) was used as a negative control and reference to normalize screening plate data. mek and PTP-ER dsRNAs (green and red, respectively) were used as positive controls to verify dsRNA knockdown efficiency. (TIF) [file pbio.1001809.s001.tif]

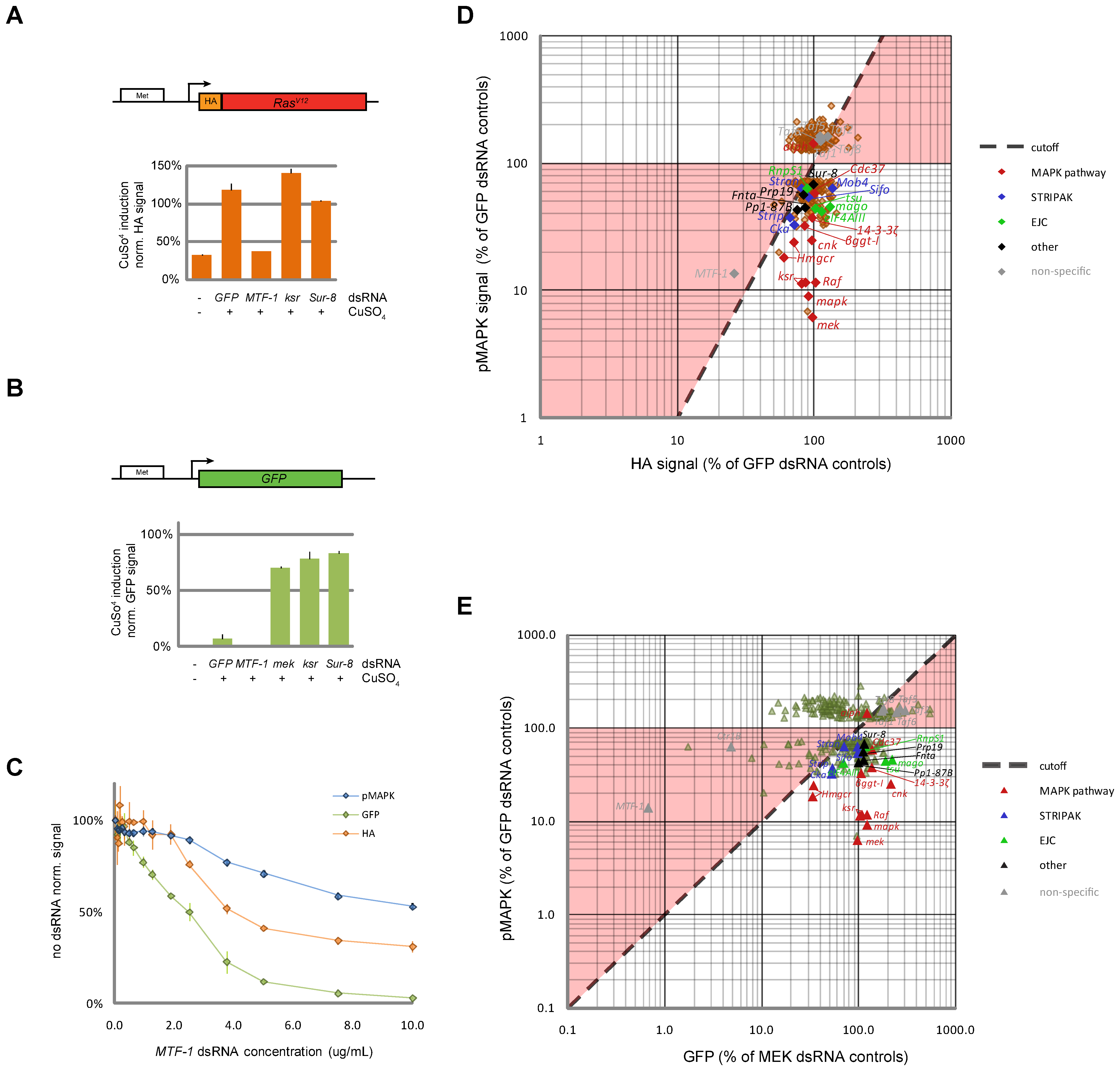

Supplement: Figure S2 — Promoter validation screens. (A–B) pMet promoter validation screen assays and control experiments. (A) RasV12 expression in pMet-HA-RasV12 stably transfected S2 cells is monitored by quantitative immunofluorescence through the use of anti-HA antibody. Values shown are the average HA signals of duplicate samples normalized to CuSO4 induced controls with no dsRNA treatment. (B) GFP expression in pMet-GFP stably transfected S2 cells is monitored by quantitative immunofluorescence. Values shown are the average GFP signals of duplicate samples normalized to CuS04 induced controls with no dsRNA treatment. (C) Variation of pMAPK, GFP, and HA signal in response to increasing amounts of MTF-1 dsRNA. Values shown are average of triplicate samples normalized to CuSO4 induced controls with no dsRNA treatment. (D–E) Promoter validation screen results (x axis) plotted against pMAPK primary screen values (y axis). The cutoff (dashed line) to identify false positive candidates with effects on promoter activity (data points in red areas) is a function of the pMAPK signal observed in the primary screen (see Text S1). MAPK regulators (red), STRIPAK (blue), EJC (green) as well as Sur-8, Pp1-87B, Fnta, and Prp19 (black) are shown. Factors known to be involved in pMet-driven expression, such as MTF-1, TBP-associated factors (Tafs), and Ctr1B are also shown (grey). (D) pMet-HA-RasV12 validation screen results. The HA signal from the validation screen (x axis) plotted against the pMAPK signal from the primary screen (y axis). The HA and pMAPK values shown are normalized to plate-specific GFP dsRNA controls. (E) pMet-GFP validation screen results. The GFP signal from the validation screen (x axis) plotted against the pMAPK signal from the primary screen (y axis). The GFP values shown are normalized to plate-specific mek dsRNA controls. The pMAPK values shown are normalized to plate-specific GFP dsRNA controls. (TIF) [file pbio.1001809.s002.tif]

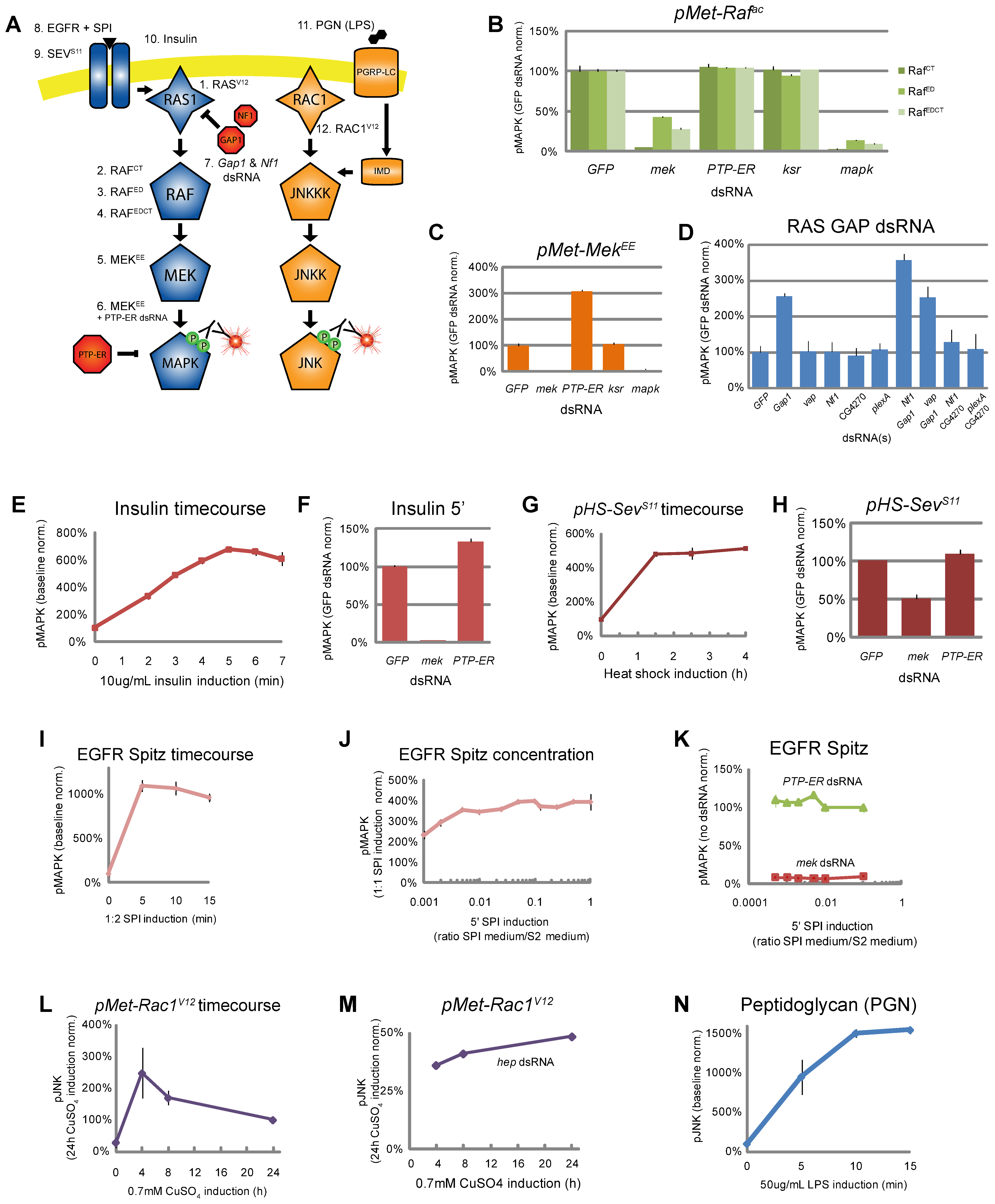

Supplement: Figure S3 — Secondary screens. (A) MAPK and JNK pathway models depicting the secondary screen assays used in this study. See Text S1 for screen descriptions. (B–N) Secondary screen control assays. Values shown are duplicate sample averages normalized to GFP dsRNA treated controls unless otherwise indicated. (B–K) are MAPK activation assays with a pMAPK readout. (L–N) are JNK pathway assays with a pJNK readout. (B) RAF-based MAPK activation: Values shown are for pMet-rafED, pMet-rafCT, and pMet-rafEDCT stably transfected cell lines treated with the indicated dsRNAs and induced with CuSO4 for 24 h. (C) MEK-based MAPK activation: dsRNA treated pMet-mekEE stably transfected cells were induced with CuSO4 for 24 h. (D) RAS GAP RNAi-based MAPK activation: S2 cells were treated with dsRNAs targeting the indicated RAS GAPs or predicted RAS GAPs. Values are normalized to untreated control samples. Combined knockdown of Gap1 and Nf1 produced the highest pMAPK activation and was used in the secondary screen assay. (E and F) Insulin-based MAPK activation. (E) Insulin induction time course: The values shown are baseline normalized signal averages from single well samples of insulin treated S2 cells induced for the indicated times. (F) The signals shown are the average from dsRNA-treated single well samples of insulin induced S2 cells induced for 5′. (G and H) SEVS11-based MAPK activation. (G) Heat shock induction time course of pHS-sevS11 stably transfected S2 cells. Samples are induced for 30 minutes at 37°C and incubated at 27°C for the indicated times before sample preparation. Values shown are normalized to a non-induced control. (H) dsRNA-treated pHS-sevS11 cells were induced with a 30′ minutes heat shock at 37°C followed by a 2.5 h incubation at 27°C. (I–K) EGFR-based MAPK activation assays. (I) Induction time course was performed on pMet-Egfr stably transfected cells induced with 1∶2 supernatant from pMet-Spi cells. Values are normalized to non-induced controls. (J) Calibration of Spi [file pbio.1001809.s003.tif]

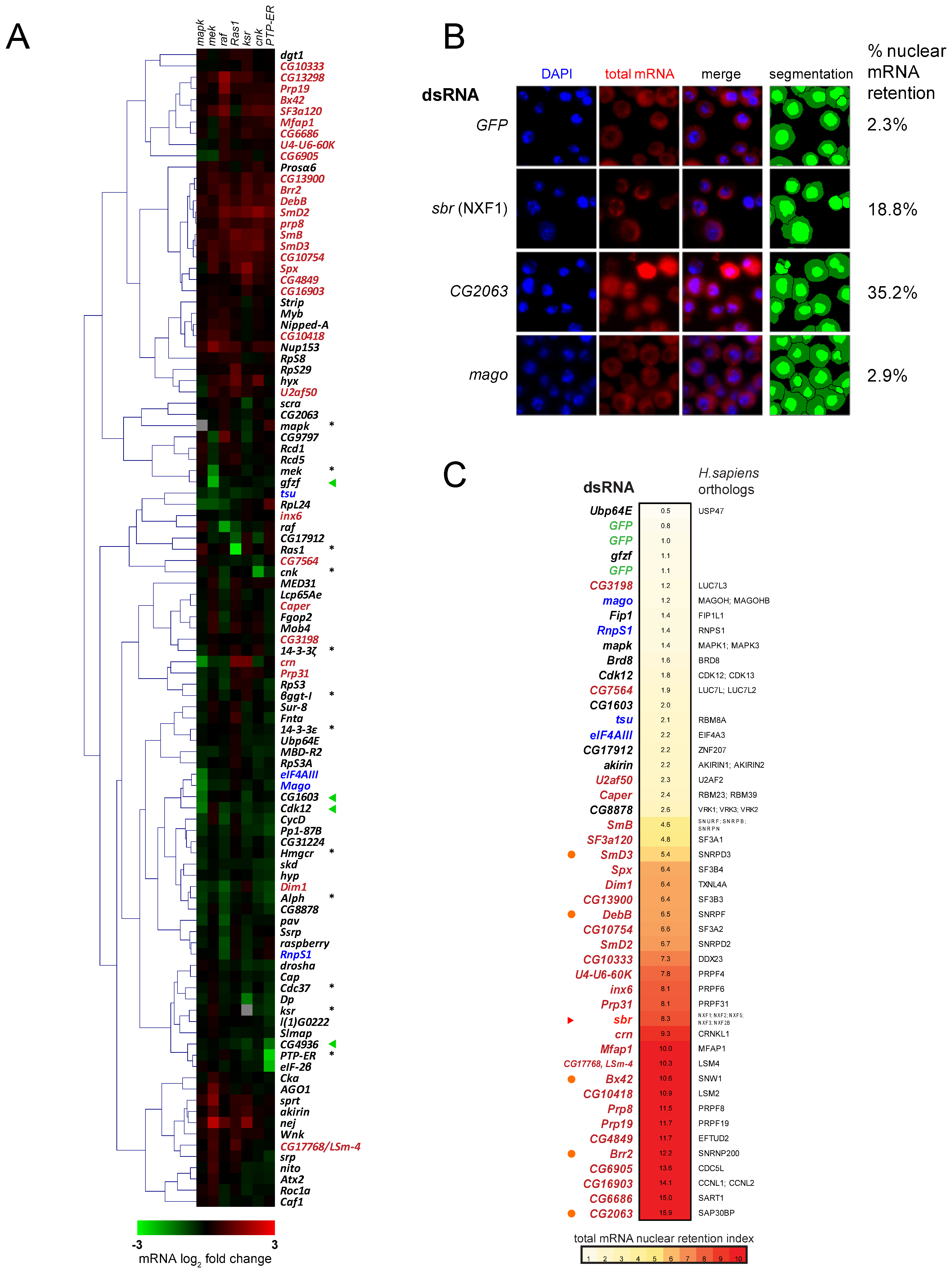

Supplement: Figure S4 — qPCR screen. (A) Unsupervised hierarchical clustering of qPCR screen results. SYBR green qPCR assays were used to assess the levels of RAS/MAPK pathway transcripts (top labels) following depletion of the indicated RasV12 screen candidates (labels, left). The transcript levels are presented as log2 transformed ratios of GFP dsRNA treated controls. RAS/MAPK control dsRNAs are indicated by asterisks. The gfzf dsRNA has the closest profile to the mek control dsRNA, with both reagents causing a reduction in mek levels. PTP-ER and CG4936 also have very similar profiles and cause a reduction in PTP-ER transcript levels. As we previously reported, the EJC components (blue) mago and eIF4AIII cause an observable decrease in mapk transcript levels. However, the knockdown of tsu and RnpS1, which have a weaker impact on pMAPK and MAPK levels, does not cause a readily observable change in mapk. Only CG1603 and Cdk12 had similar profiles to the two stronger EJC components, causing a decrease in mapk transcript levels. The Fip1 dsRNA results in the qPCR screen were not valid, but Fip1 dsRNA was re-tested in the confirmation qPCR experiment presented in Figure 2 and found to have a similar profile to the two other factors. Most of the splicing factors—other than the two EJC components—were not found to cause changes in the levels of mapk or any of the other RAS/MAPK transcripts. (B) Total mRNA FISH performed using an oligo-dT Cy3-labeled probe. The proportion of cells displaying nuclear retention was evaluated by performing segmentation and scoring the overlap of the FISH signal with a DAPI nuclear stain. dsRNA targeting sbr, the homolog of the mammalian nuclear RNA export factor 1 (NXF1), and CG2063, a factor previously linked to mRNA export, both caused an increase in nuclear retention. In comparison mago dsRNA did not visibly alter total mRNA retention. (C) Total mRNA export screen results. A subset of 44 candidates were tested to evaluate their impact on total mRNA export: these [file pbio.1001809.s004.tif]

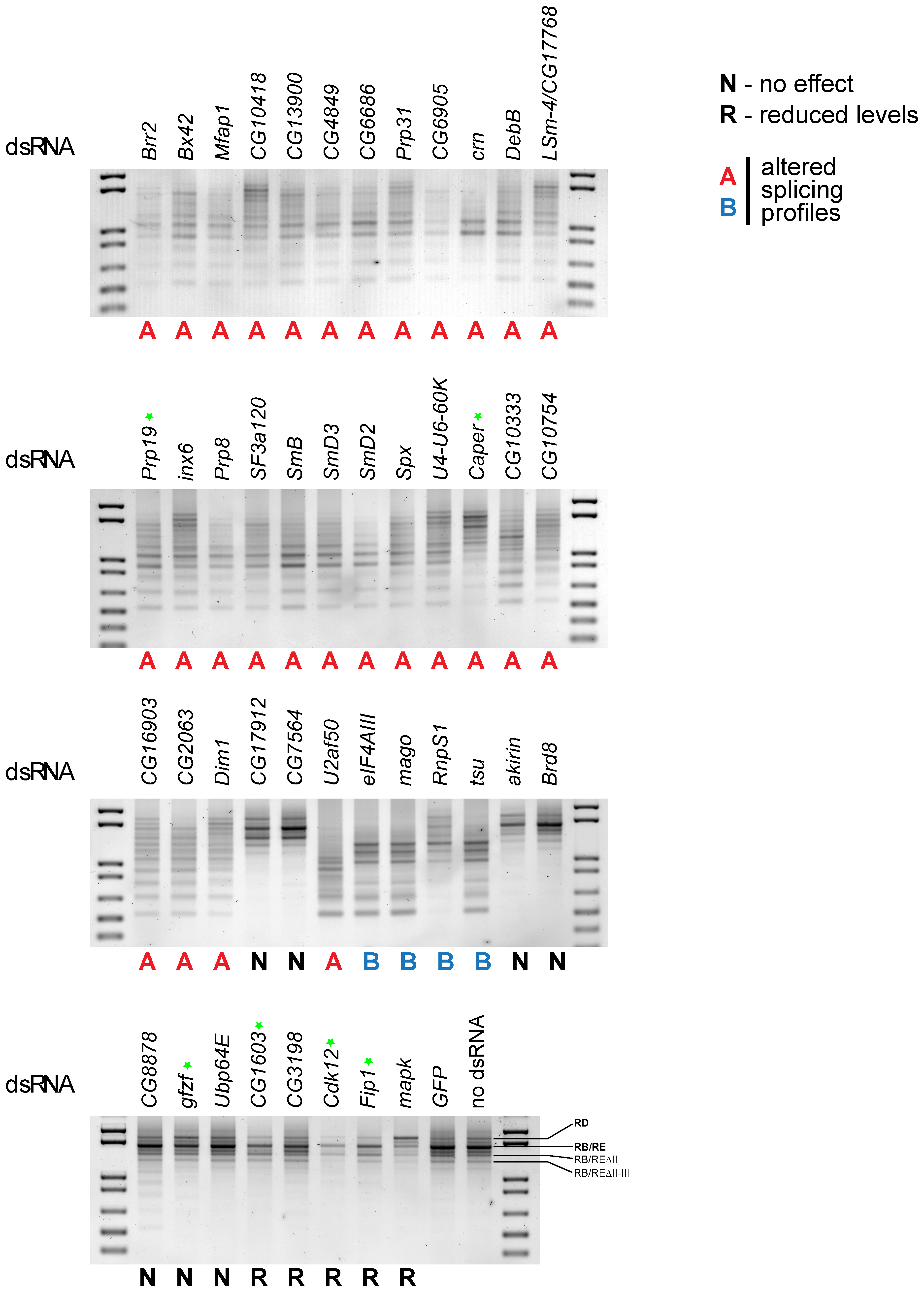

Supplement: Figure S5 — RT-PCR screen. An RT-PCR assay encompassing the entire mapk RB, RD, RE, and RF transcripts was used to evaluate the impact on the distribution of mapk transcript isoforms. The same subset of 44 hits selected for the mRNA export screen were also tested here. A majority of factors tested caused a change in the mapk RT-PCR profile when compared to GFP dsRNA treated or untreated controls. The altered profiles were grouped into two categories (A and B) on the basis of similarities in the sizes of the bands observed. Most splicing factors in the set produced an “A” type profile while EJC factors produced a different “B” type profile. Other candidates causing no obvious change in product size, but causing a reduction in band intensity are labeled “R.” Those causing no observable change are labeled “N.” The labels on the right of the bottom panel refer to mapk transcript isoforms that correspond to the bands visible on the gel. Candidates featured in the manuscript are labeled with a green star. (TIF) [file pbio.1001809.s005.tif]

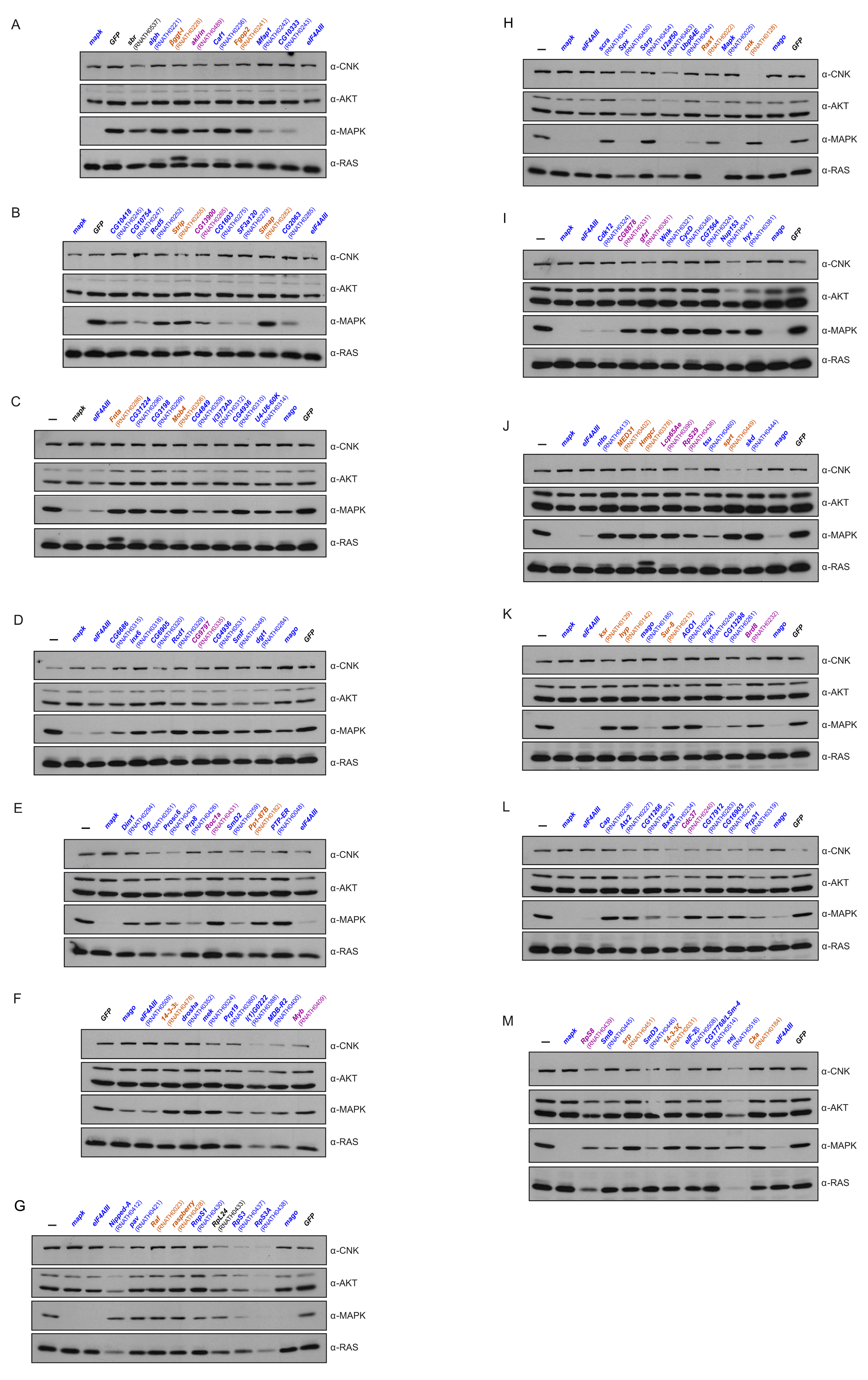

Supplement: Figure S6 — Immunoblot screen. (A–M) Western blot analyses of S2 cells treated with the indicated dsRNAs. Endogenous levels of CNK, AKT, MAPK, and RAS were monitored using specific antibodies. A negative (GFP dsRNA) and two positive (mago and eiF4AIII dsRNAs) controls were included in this experiment. (TIF) [file pbio.1001809.s006.tif]

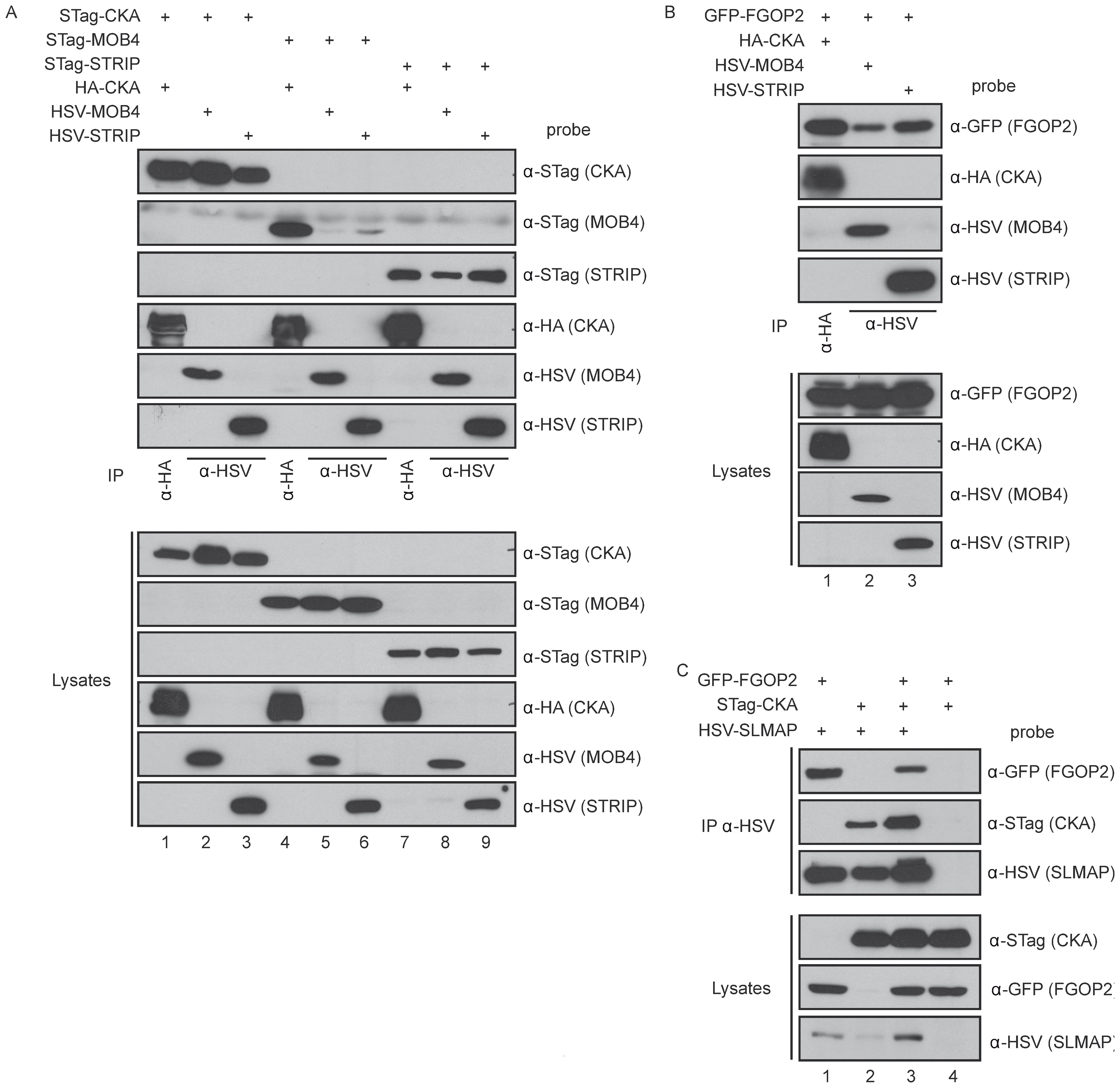

Supplement: Figure S7 — Co-immunoprecipitation of Drosophila STRIPAK complex components. S2 cells were transfected with expression plasmids carrying cDNAs encoding the fusion proteins indicated at the top of (A, B, and C). Cell lysates were immunoprecipitated with the indicated antibodies (bottom of upper panels in (A and B), and left of upper panels in (C)). The immunoprecipitates and equal amounts of cell lysates (normalized for total protein content) were fractionated by SDS-PAGE and immunoblotted with the antibodies indicated at the right (A–C). The tagged STRIPAK complex protein shown in each panel is indicated in parentheses. None of the STag fusion proteins or GFP-FGOP2 are present in α-HA or α-HSV immunoprecipitates when these fusion proteins are expressed alone, and GFP does not co-immunoprecipitate with HA-CKA, HSV-MOB4, HSV-STRIP, or HSV-SLMAP (unpublished data). (TIF) [file pbio.1001809.s007.tif]

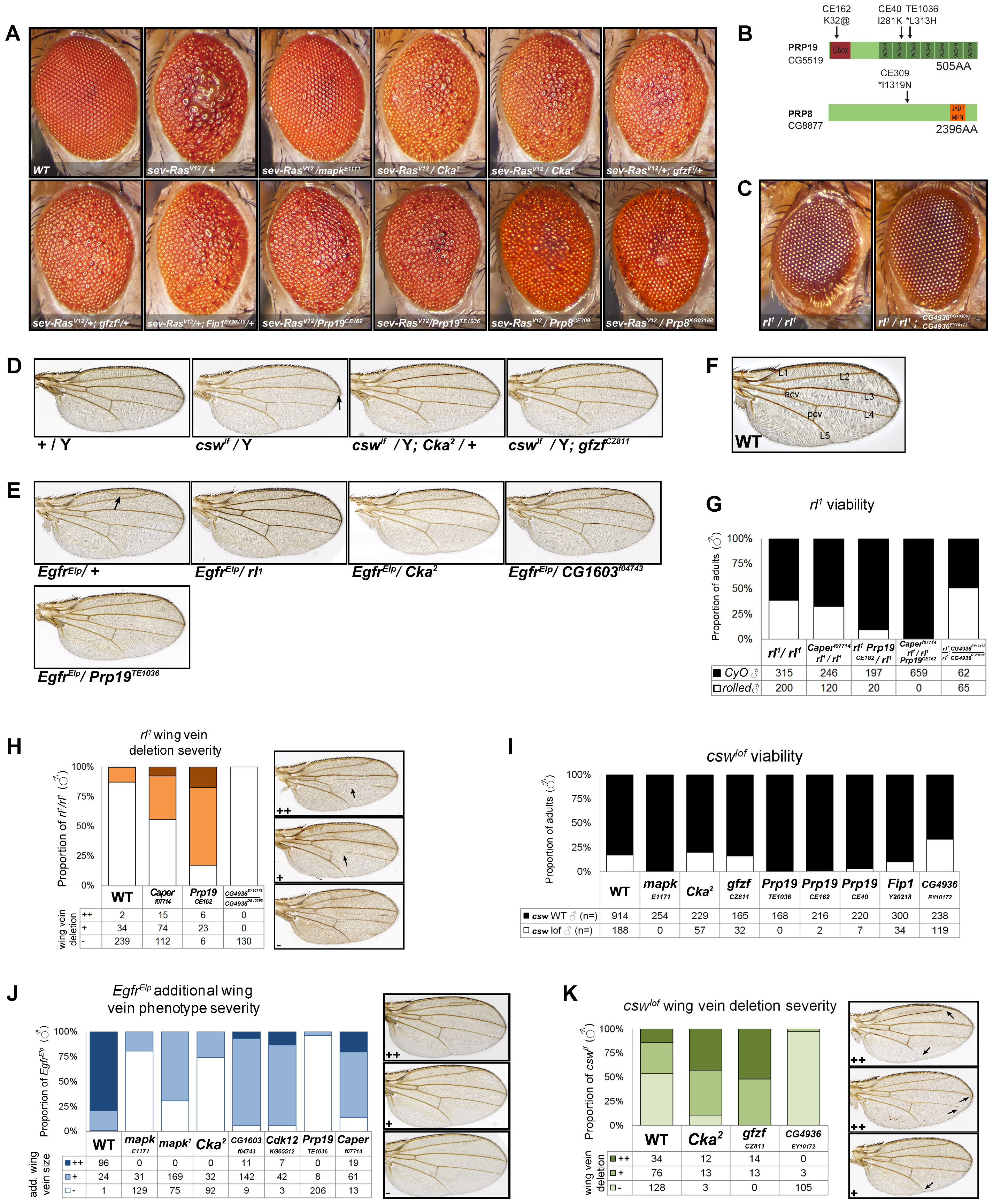

Supplement: Figure S8 — Additional genetic interaction data. (A) RasV12 genetic interaction data for additional alleles of Cka, gfzf, and Prp19 as well as alleles of Fip1 and Prp8. (B) Details of molecular lesions present in the three alleles of Prp19 and the Prp8CE309 allele identified in a cnk dominant negative genetic screen (CB, ML, MS, and MT, unpublished data) and used in our genetic validation experiments. A protein map of PRP19 and PRP8 showing the amino acid changes found in the Prp19 and Prp8 alleles is presented with “*” indicating residues conserved in humans. The mutation in Prp8CE309, in addition to causing an amino acid change, is located on the second residue of a 3′SS (T4088A) and may impact splicing of Prp8. (C) Additional rl1 rough-eye phenotype genetic interactions for CG4936. Trans-heterozygous CG4936DG10305/CG4936EY10172 suppresses the weak rough eye phenotype of rl1 homozygotes. Eye size is also slightly restored. (H) Representative wings for cswlf hemizygous males scored in (K). The deletion of the distal end of the L2, L3, and/or L5 vein is a frequently observed phenotype in a cswlf background. The occurrence, number, and severity of this deletion are more pronounced in Cka2 and gfzfCZ811 heterozygous backgrounds. (E) Representative wing images for the EgfrElp males scored in (J). The additional wing vein near the extremity of the L2 vein (arrow) is characteristic in EgfrElp flies. Wings displaying a suppressed phenotype (reduced frequency and length of the extra vein material) are shown for Cka2, CG1603f04743, Prp19TE1036, and mapk/rl1 (positive control) heterozygotes. (F) Image of a wild-type fly wing with labels indicating the location of the five wing veins (L1–L5) and of the anterior (acv) and posterior (pcv) cross-veins. (G) Proportion of rl1/rl1 males to rl1/CyO males observed following a rl1/CyO X rl1/CyO cross. The wings of rl1/rl1 flies have a “rolled” phenotype; they are slightly curved downwards along the anterior-posterior axis. rl1/CyO flies display a [file pbio.1001809.s008.tif]

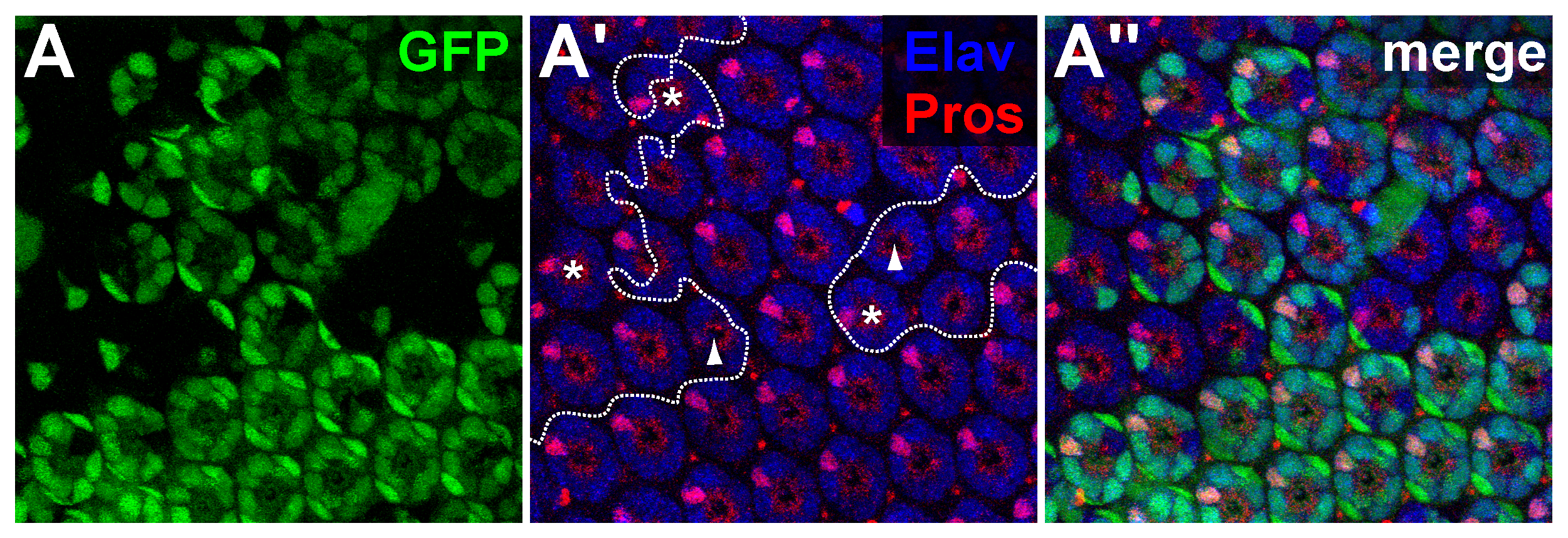

Supplement: Figure S9 — Cka is important for photoreceptor development. (A) Cka2 mutant clones in pupal eye discs dissected 42 h APF are marked by the absence of GFP (A). The differentiated R7 photoreceptor is marked by the overlapping Elav and Pros stainings (in purple, A′ and A″). In Cka2 clones, ommatidia lacking the R7 photoreceptor are marked with a white arrow. We also noted some displaced R7 photoreceptors (R7s outside the focal plane are marked with white arrowheads), and rotated ommatidia (white asterisks) in the Cka2 clones. (TIF) [file pbio.1001809.s009.tif]

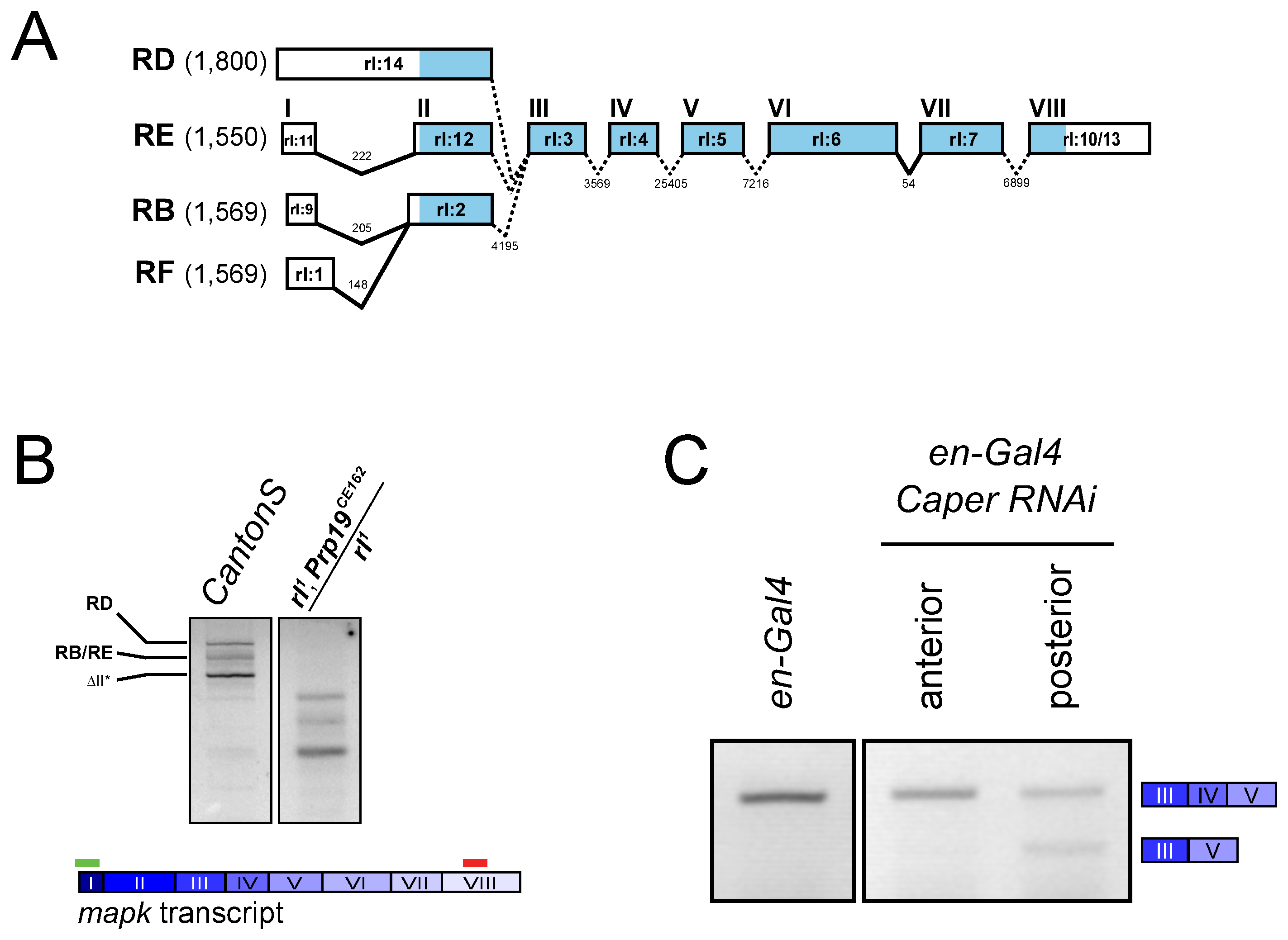

Supplement: Figure S10 — In vivo evidence for alternative splicing of mapk . (A) Schematic representation of the mapk splice isoforms from Figure 8. (B) The RT-PCR assay spanning the whole mapk transcript was used to detect splicing changes in adult flies. Samples were prepared from five adult flies of the indicated genotypes. Escapers homozygous for rl1/rl1 and also carrying the Prp19CE162 mutant displayed splicing changes compared to a CantonS wild type control strain. The products from this experiment were not verified by sequencing though they were similar to those observed in S2 cells upon Prp19 knockdown (the lower sized band in the WT control is assumed to be due to skipping of exon II, based on our S2 cell data). rl1 homozygous flies have a reduced amount of mapk though the size of the RT-PCR product is unchanged (not shown). (C) mapk splicing is altered in larval wing imaginal discs following Caper knockdown. A RNAi construct targeting Caper was expressed in the posterior segment of wing imaginal discs using an engrailed-GAL4 driver. Imaginal discs from 3rd instar larvae were microdissected to separate the posterior (control) and anterior (Caper knockdown) segments. The RT-PCR assay spanning exons III–V was used on extracts from both samples to evaluate inclusion of exon IV. All Prp19 RNAi tested with the Engrailed-Gal4 driver caused a high rate of lethality and escapers that could be recovered were of reduced size and were not found to contain wing discs. (TIF) [file pbio.1001809.s010.tif]

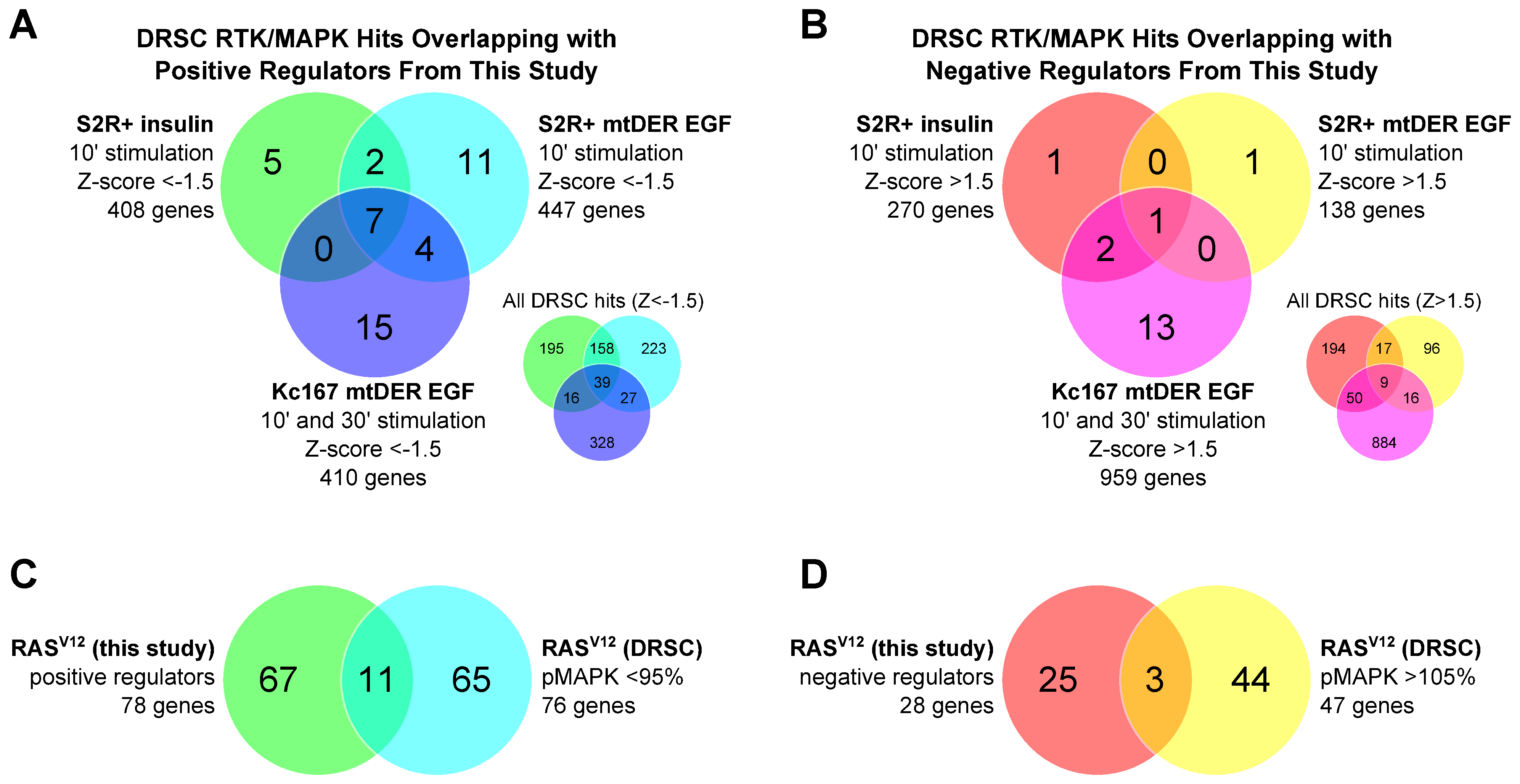

Supplement: Figure S11 — Comparison with previous RTK/MAPK RNAi screens. (A and B) Overlap of validated positive (A) and negative (B) regulators of RASV12 identified in this study with positive and negative regulators reported in three Drosophila RNAi Screening Center (DRSC) RTK/MAPK screens [37],[62]. The diagrams include all genes reported as Insulin and EGF modulators (both in S2R+ and Kc cells) with a Z-score ±1.5 (986 positive and 1,266 negative regulators in total). The large Venn diagrams display hits in the three DRSC screens overlapping with hits reported here. The total hits reported in the DRSC screens are displayed in the small diagrams. Reported gene identifiers were first updated to the current annotation before comparison was performed. (C and D) Overlap of validated positive (C) and negative (D) regulators of RASV12 identified in this study with 331 validated hits from the DRSC S2R+ insulin screen that were tested in a RASV12 secondary screen [62]. 123 hits from the DRSC screen were reported to modulate pMAPK signal by over ±5% and were considered as hits for the purpose of this comparison. (TIF) [file pbio.1001809.s011.tif]
