# Supplementary material for: A Functional Screen Reveals an Extensive Layer of Transcriptional and Splicing Control Underlying RAS/MAPK Signaling in Drosophila
Source: PLoS Biol. 2014 Mar 18;12(3):e1001809. doi: 10.1371/journal.pbio.1001809 (PMC3958334; doi:10.1371/journal.pbio.1001809)
Supplement: Table S2 — GO term enrichment. GO terms found to be enriched in our set of validated hits using the Flymine [129] GO enrichment function. p-Values are calculated using Benjamini and Hochberg multiple hypothesis test. (DOCX) [file pbio.1001809.s013.docx]

#### Table S2. GO Term Enrichment

| **GO accession** | **GO term** | **genes** | **p-value** |
| --- | --- | --- | --- |
| GO:0006397 | **mRNA processing** | 35 | 1.1E-24 |
| GO:0008380 | **RNA splicing** | 33 | 1.9E-24 |
| GO:0006396 | **RNA processing** | 37 | 1.6E-23 |
| GO:0022008 | **neurogenesis** | 51 | 4.2E-20 |
| GO:0030154 | **cell differentiation** | 59 | 3.9E-18 |
| GO:0007049 | **cell cycle** | 38 | 2.8E-16 |
| GO:0010467 | **gene expression** | 56 | 8.7E-14 |
| GO:0000226 | **microtubule cytoskeleton organization** | 26 | 5.6E-13 |
| GO:0006996 | **organelle organization** | 35 | 3.2E-07 |
| GO:0009790 | **embryo development** | 19 | 1.1E-04 |
| GO:0051716 | **cellular response to stimulus** | 32 | 8.2E-04 |
| GO:0051298 | **centrosome duplication** | 7 | 1.8E-03 |
| GO:0006974 | **response to DNA damage stimulus** | 11 | 3.1E-03 |
