# Supplementary material for: A Functional Screen Reveals an Extensive Layer of Transcriptional and Splicing Control Underlying RAS/MAPK Signaling in Drosophila
Source: PLoS Biol. 2014 Mar 18;12(3):e1001809. doi: 10.1371/journal.pbio.1001809 (PMC3958334; doi:10.1371/journal.pbio.1001809)
Supplement: Text S1 — Notes on the exon junction complex and supplemental methods. Document containing additional notes on the exon junction complex as well as an extended description of the experimental methods used in this study. (DOCX) [file pbio.1001809.s020.docx]

# A Functional Screen Reveals an Extensive Layer of Transcriptional and Splicing Control Underlying RAS/MAPK Signaling in Drosophila

# TEXT S1

## ADDITIONAL NOTES ON THE EXON JUNCTION COMPLEX

The exon junction complex was originally identified as a regulator of RAS/MAPK signalling in the RNAi screen described in this manuscript. The characterization of the EJC’s function in the regulation of *mapk* splicing is described in detail in a previous paper from our group[1] as well as in another study[2]. In this study, we chose to focus on other hits from the screen and on the splicing factor group in particular. Experiments involving EJC components are used to highlight the differences between the previously descrived EJC function and the novel regulation of *mapk* splicing described here.

Recently, a new EJC associated factor, CWC22, was identified[3-5]. CWC22 is proposed to act as a link between EIF4A3 to spliceosome. Interestingly, CWC22 is also a splicing factor[5] which fits in well with our initial observation that the EJC components can also act on splicing. This also ties in nicely with a recent paper reporting a comprehensive EJC-RNA and EJC-protein interactome[6]. Namely, binding of the EJC to non-canonical sites and assembly into higher order structures that include SR factors is observed, which would be consistent with a function in alternative splicing.

The *Drosophila* homologue of CWC22, *ncm*, while not a hit in our screen, was very close to the cutoff threshold. Moreover, *ncm*, like *Prp8*, was found to enhance the *aos* induced small wing phenotype[7], suggesting that it also acts on RTK/MAPK signalling.

## SUPPLEMENTAL METHODS

### Genome-wide dsRNA Library

The dsRNA library was generated from dsDNA templates purchased from Open Biosystems (Huntsville, AL; http://www.openbiosystems.com). The Expression Arrest *Drosophila* RNAi Library (versions 1 and 2) covers most of the protein-coding genes in the annotated *Drosophila* genome and is described in detail in[8]. The 15880 individual dsRNAs were generated at the IRIC high throughput screening facility by *in vitro* transcription using T7 RNA polymerase. dsRNA probe concentration was assessed using RiboGreen RNA reagent (Invitrogen). Transcription was repeated when necessary. Products were diluted to an average concentration of 0.2μg/μl and purified on filter plates (Millipore). Additionally, 25% of the dsRNAs were verified on gel (E-Gel, Invitrogen). The library is stored in sealed 384-well plate aliquots at -30°C.

### Primary Screen Assay

The Z’-factor[9] was used to calculate assay suitability for high throughput screening considerations. This calculation was done using *GFP* dsRNA negative controls and *mek* and *PTP-ER* dsRNA positive controls for suppressors and enhancers, respectively. The calculated Z’ was 0.643 for *mek* and 0.175 for *PTP-ER*. This meant that the assay was highly suitable for detection of suppressors, but was not as robust in the case of enhancers. This lower Z’ reflects the slightly larger variation in the calculated signals of *PTP-ER* controls as well as the lower difference between *GFP* and *PTP-ER* control means (Figure S1B). The lower reliability in the case of enhancers may be caused by the strong pathway activation state due to the overexpression of RAS^V12^. The difference between the negative control and enhancer populations would therefore not be as pronounced because pathway activity is already at a heightened state.

For the primary and promoter validation screening steps, the library dsRNAs as well as control dsRNAs were arrayed in bar-coded 96 well clear flat bottom cell culture microplates (Corning) by a Biomek FX (Beckman) in a sterile culture hood. The cells were then plated at a concentration of 50,000 cells per well using a multi-channel pipettor (BioHit). After a three day 25°C incubation period, 0.7mM CuSO_4_ (diluted Schneider medium) was added using a multi-channel pipettor and cells were incubated for another 24h, for a total of four days of dsRNA incubation. Following incubation and CuSO_4_ induction, the subsequent staining steps were performed using an automated procedure developed on an integrated roboticized platform. Briefly, a Biomek was used to resuspend and transfer cells to a second set of identical ConA coated plates. After a 1h incubation step, cells were fixed in a 4% paraformaldehyde solution for 15’. A PBT (PBS Triton 0.2%) BSA 0.2% solution was used to wash, permeabilize and block cells using a washing station (BioTek ELx405). PBT BSA 2% was used as a solution for incubation in both anti-pMAPK, as well as a mixed anti-mouse Alexa555 antibody and DAPI solution. These reagents were distributed using a Multidrop (Thermo). Following the staining procedure, cells were mounted in Mowiol (9.6% PVA, 25% Glycerol) and sealed using adhesive plate covers. The plates were read using a wide field inverted microscope (Axiovert, Zeiss) equipped with a motorized XY stage as well as an objective turret mounted on a mobile Z axis. Metamorph (Molecular Devices) software was used to automate autofocus and image capture procedures. Image were acquired at a 20X magnification at a rate of two fields per well (primary screen) or five fields per well (primary screen confirmation step and validation step).

To control for problems in cell resuspension and transfer, the initial plates were stained with Methylene Blue in order to detect cases of increased cell adhesion. Stain was then dissolved in DMSO and OD 600nm was measured to approximate cell number. The Methylene Blue images and values are available on the IRIC *RNAi* database.

Quantification was carried out using the Cell Scoring application in Metamorph. Plate-specific background values were subtracted to all images. The integrated intensity from Alexa555 positive cells was divided by the total DAPI cell nucleus count to obtain an average measure of pMAPK signal per cell. The values of the *GFP* negative control wells were used for plate data normalization.

Cutoffs for our primary dataset were set at -0.45 (73.2%; suppressors) and 0.34 (126.8%; enhancers) log_2_ ratio of the *GFP* dsRNA control average (Figure 1A). By comparison, the standard deviation of the dsRNA set was 0.21 and that of the *GFP* negative controls was of 0.09. Out of the 1322 *GFP* control well values, one is outside the cutoff margins (Figure S1C), allowing to estimate the rate of false positives due to assay noise at 0.0756%. Out of 1598 positive controls tested, only 6 positive controls, 1 *mek* and 5 *PTP-ER*, resulted in false negatives (Figure S1C). Accordingly, the global false negative rate for the assay could therefore be estimated at 0.375%, or more specifically, at 0.125% for suppressors and at 0.626% for enhancers. The selected cutoff value for suppressors is over 2x the standard deviation of the dsRNA set and 4.5x the GFP standard deviation. The cutoff for enhancers was more permissive (1.6x and 3.9x) in order to include more enhancers in our set.

Following the genome-wide screen, probes that scored as preliminary hits were re-tested to confirm the primary screen data. Only those probes were retained whose average signal (of the primary and primary confirmation screen values) was outside the cutoffs. Of the confirmed hits, additional candidates were also eliminated based on cell clustering phenotypes (visually scored) or low cell count values, which interfere with proper quantification.

### Validation Screens

Because MTF-1 (Metal response element-binding Transcription Factor-1), a key transcription factor involved in Metallothionein promoter (*pMet*) activity[10], was also amongst the strongest suppressors identified (Table S1), we conducted a first series of validation assays using *pMet-GFP* and *pMet-HA-Ras^V12^* cell lines to eliminate false positives that influenced *pMet* activity. This was done by measuring the GFP and HA signals and comparing them to the primary screen pMAPK signal. Because these assays relied on the production of a protein signal, they also allowed for the elimination of hits that affected basic cellular machineries involved in protein synthesis such as mRNA export and translation. Depletion of *MTF-1*, but not of RAS/MAPK pathway components, caused reduction in GFP and HA levels, allowing to identify false positive hits of this type (Figure S2). Both validation screens were carried out using a similar protocol to the one used in the primary screen (see above).

We observed that pMAPK levels (induced by *pMet-Ras^V12^*) was less sensitive to disruption of promoter activity than GFP or HA levels (induced by *pMet-GFP* or *pMet-HA-Ras^V12^*) (Figure S2). Therefore, we used a cutoff that was a function of the primary screen signal for the GFP and HA screens:

$$GFP screen cutoff= x$$

$$HA screen cutoff= {10}^{2\log_{10} x-2}$$

Where *x* is the normalized signal from the GFP or HA screen. The calculated cutoff is applied to the primary screen result. Candidates with primary screen pMAPK value below the cutoff (for suppressors) or above the cutoff (for enhancers) are retained (Figure S2). This result indicates that there is no effect on promoter activity or that it is not significant enough to account for the observed variation in pMAPK. 233 hits passed these *pMet* validation steps (Table S1).

In the second validation step (Figure S1A), we addressed potential dsRNA off-target effects. We synthesized new, non-overlapping, dsRNA probes targeting these 233 genes and retested them in the RAS^V12^ assay in four replicates, but in conditions otherwise similar to those described for the primary assay. Importantly, these probes were designed to be devoid of off-predicted target sequences using either the E-RNAi (http://www.dkfz.de/signaling/e-rnai3/)[11] or SnapDragon (http://www.flyrnai.org/cgi-bin/RNAi_find_primers.pl) dsRNA design tools. 101 probes from this validation step recapitulated the effect of the primary probes on pMAPK levels.

### Secondary Screens: Functional Assays

The procedure for conducting the secondary screens was similar to the one described for the primary and validation steps, except that the protocol was not automated. In total, 13 secondary screens were performed using the secondary probe set that had passed the validation step criteria. 12 signaling screens (Figure S3A) and a second *pMet-GFP* promoter validation step were performed. The same dsRNA probe set was used for the protein and transcript level screens as well as the bulk mRNA export screen. For the secondary RAS^V12^ assay, the data from validated probes (in the second validation step) was used here and considered as secondary screen data). Validation of the 11 other secondary assays is shown in Figure S3 along with a description of the induction procedures. All dsRNA incubations were performed for four days in duplicate or larger replicate sizes.

As in the primary screen assay, automated microscopy and image analysis were used for quantification and secondary screen and functional assay data was also normalized to plate-specific dsRNA controls (*GFP* dsRNA, or *mek* dsRNA in the case of the *pMet-GFP* assay).

### qPCR and RT-PCR

For the qPCR secondary screen, triplicate S2 cell culture replicates were lysed using a Cells-to-cDNA™ (Life technologies) lysis buffer following manufacturer guidelines. The qPCR was performed in two steps using a High Capacity cDNA Reverse Transcription Kit (Life technologies) for reverse transcription and SYBR^®^ Green PCR kit (Life technologies) for DNA quantification. When selecting hits for follow-up analysis, we considered significant changes in transcript abundance greater than +/- 1 log_2_ fold change with a p-value < 0.005 (unpaired two-tailed Student’s t-test). We eliminated dsRNAs that significantly altered the levels of more than one transcript. We performed confirmation experiments only on the candidates for which the modulation of transcript levels was consistent with the functional effect (ex.: *Cdk12* dsRNA decreases *mapk* transcript levels and also supresses RAS^V12^-induced pMAPK). For the qPCR confirmation experiment, RNA extracts were prepared using TRIzol^®^ reagent (Life technologies) following manufacturer instructions. qPCR was then performed in technical triplicate as in the qPCR screen.

For qPCR performed on larval eye-antennal imaginal discs, RNAi constructs were expressed under the control of the same heat shock flip-out system used to generate clones for microscopy. To maximize RNAi expression, L1 larvae were heat shocked 40 minutes at 37°C. In these conditions, GFP positive clones covered the majority of dissected imaginal discs. For each sample, 3 eye-antennal imaginal discs were lysed in 5uL of Cells-to-cDNA^TM^ lysis buffer (Life Technologies), treated with DNAse and immediately used to conduct RT using SuperScript™ II. Three samples were prepared for each condition tested (biological triplicates) and qPCR was performed in technical triplicate.

For S2 cell culture RT-PCR experiments, RT was carried out on RNA samples prepared using TRIzol^®^ reagent (Life technologies). For the RT-PCR screen, we selected a subset of hits that were positioned downstream of MEK as well as predicted splicing factors positioned at other intervals. S2 cells were seeded in triplicate in 96 well plates with dsRNA. The samples were then pooled for TRIzol lysis and RNA extraction. Following this, RT-PCR was performed using a SuperScript™ II (Life Technologies) as per manufacturer guidelines. PCR was performed using Taq DNA polymerase (Life Technologies) and samples were loaded on a 2% agarose gel. All other RT-PCR experiments were performed using a High Capacity cDNA Reverse Transcription Kit (Life technologies). Cloning and sequencing of *mapk* RT-PCR products was performed as previously described in Ashton-Beaucage et al.[1]. The second exon of the RE and RB/RF (rl:12 and rl:2) as well as the first exon of RD (rl:14) were considered as exon II for purposes of calculating the exon skipping rates.

Adult fly RT-PCR experiments were performed on RNA extracted from 5 adult male flies using TRIzol. Flies were first frozen at -80°C and homogenized in TRI reagent. Following this, RT-PCR was carried out using the conditions described above.

For the RT-PCR on larval tissue, wing imaginal discs were first extracted from third instar larvae. A *UAS-GFP* under the control of the *engrailed-GAL4* promoter was used to mark the anterior segment of the wing discs. A UV lamp system (NIGHTSEA™ Fluorescence Viewing Systems) was used to visualize GFP and guide manual micro-dissection with sharpened tungsten needles. A single wing disc fragment was homogenized in 5uL of Cells-to-cDNA^TM^ lysis buffer (Life Technologies), treated with DNAse and immediately used to conduct RT using SuperScript™ II.

All primer sequences used for the qPCR and RT-PCR assays are listed in Table S8.

### FISH Total mRNA Export Screen

The fluorescence *in situ* hybridization (FISH) was adapted from Herold et al.[12]. An oligo-dT FISH probe was used to visualize polyadenylated total mRNA. Cells were transferred to 96 well concanavalin A coated glass-bottomed plates and fixed in 4% paraformaldehyde (4% PFA in PBS) for 10 minutes. The PFA was then quenched in PBS-Glycine 1.25M for 5 minutes and PBS-triton 0.5% (10 min) was used for permeabilization. PBS wash steps preceded and followed each of these steps. Next, samples were incubated in pre-hybridization buffer (2X saline-sodium citrate[SSC; 0.3M sodium chloride, 30mM trisodium citrate, pH 7.0], 20% formamide, 0.2% BSA, 1mg/mL yeast tRNA) for 15 min at 37°C, then hybridized with the oligo-dT probe for 2h in hybridization buffer (2X SCC, 20% formamide, 0.2% BDS, 1mg/mL yeast tRNA, 10% dextran sulfate, 0.1 pmol/uL Cy3-oligo-dT) at 37°C. Following hybridization, samples were incubated twice in 2X SCC 20% formamide and twice in 2X SCC for 5 minutes at 42°, once in 1X SCC for 5 min, once in DAPI (DAPI 0.1 µg/mL in PBS) and washed once in PBS before being coated in Mowiol.

Cells were imaged by automated microscopy using an Operetta high content imaging system (PerkinElmer). Segmentation and signal quantification was performed using Harmony® software (PerkinElmer).

### The IRIC RNAi Database

Images and quantitative data were imported into the IRIC RNAi database (www.bioinfo.iric.ca/iricrnai/). IRIC RNAi is a custom SQL database intended for use in storing and referencing RNAi screen data conducted at the Institute for Research in Immunology and Cancer (IRIC). The screening data described in this paper is made available at *IRIC* *RNAi* as a web resource accompanying this paper in the “*Ras^V12^* pMAPK” project section. Information on the screening assays described in this study is also made available. The database allows access to specific experimental results, probe sequence and genomic information. It is also possible to view the automated microscopy images used for signal quantification. To find information relating to a specific gene or RNAi probe, a gene and probe identifier search can be performed. A genome browser allows the user to visualize dsRNA probe localization relative to gene sequence.

Part of the image analysis was also conducted through the IRIC RNAi database. Out-of focus images from the primary screen were filtered out using a custom algorithm. The algorithm calculates the average frequency of the set of DAPI images using a two-dimensional Fourier transform. A threshold was set to sort out blurred images with lower average frequencies. A manual quality control and visual inspection was also performed using the plate overview and image viewing functions. All negative controls and primary hits were inspected visually to sort out errors due to obvious staining artefacts.

### Additional information for epistasis analysis

We used a modified *Pearson’s Uncentered Correlation* with weight (***w***) values in order to correct for the fact that data from one RAS-based screen, three RAF-based screens and two MEK-based screens were being used to position our candidates. *w*_1_ (for 1. RAS^V12^) was set to 3, *w*_2_, *w*_3_, and *w*_4_ (2. RAF^CT^, 3. RAF^ED^, 4. RAF^EDCT^) to 1, *w*_5_ (5. MEK^EE^) to 1 and *w*_6_ (6. MEK^EE^ + PTP-ER dsRNA) to 2. Thus data from RAS, RAF and MEK contribute equally to the calculated correlation *r*. Screen #6 (MEK^EE^ + *PTP-ER* dsRNA) was given a higher weight than screen #5 (MEK^EE^) based on the our observation that the addition of *PTP-ER* dsRNA produced a higher signal induction (Figure S3C) and that modulation of this signal was more robust than for MEK^EE^ alone (data not shown).

Candidates with poor correlation (-0.5 < *r <* 0.5) in all three profiles were not assigned an epistasis interval. Candidates with similar *r* values for all three profiles were marked as ambiguous based on a confidence score:

$$c= \left| r_{1}-\frac{r_{2}r_{3}}{2} \right|$$

Where *c* is the epistasis confidence score and *r*_1_, *r*_2_ and *r*_3_ the first, second and third highest correlation values. Thus, candidates for which the highest correlation value (*r*_1_) was close to the average of the other two calculated correlation values (*c* < 0.2) were labeled as ambiguous. See Table S3 for the full set of correlation values and confidence scores.

Both unsupervised clustering analyses presented if Figures 3A were performed on log_10_ transformed secondary screen values using a hierarchical clustering function and *Pearson’s Uncentered* distance metric in the MeV application (http://www.tm4.org/mev/)[13]. The 3D graphical representation shown in Figure 2C was assembled using OriginLab (<http://www.originlab.com/>).

### Specificity score

Four datasets were used to calculate the specificity score: 1) secondary functional screen data, 2) mRNA export screen data, 3) hit occurrence in previously published RNAi screens and 4) the western blot secondary screen. Scores assessed from these three sources were added to obtain the final specificity score (with a higher specificity score corresponding to a less specific hit).

Secondary screen data was analyzed as follows. Specificity with regards to JNK signaling and effects on *pMet* promoter activity were both assessed. As in the epistasis analysis, *Pearson’s Uncentered Correlation* was used to assess correlation with predetermined specificity profiles in both cases. The correlations were calculated on normalized secondary screen Log_10_ transformed values:

$$r\left( x,y \right)=\frac{\sum_{i=1}^{n} {(x}_{i}y_{i})}{\sqrt{\sum_{i=1}^{n} x_{i}^{2}}\sqrt{\sum_{i=1}^{n} y_{i}^{2}}}$$

Where *r* is the correlation value[-1,1]. The secondary screen values *x* and specificity profiles ***y*** are specified below.

For JNK specificity assessment *x* corresponds to data from screens # 1, 11 and 12 (1. RAS^V12^, 11. PGN and 12. RAC1^V12^). Correlation with 4 possible profiles was assessed:

**PGN-RAC1^V12^** =[1 1 1] (score: 2; similar variation in RAS^V12^, PGN and RAC1^V12^)

**PGN** =[1 1 0] (score: 1; similar variation in RAS^V12^ and PGN only)

**RAC1^V12^** =[1 1 0] (score: 1; similar variation in RAS^V12^ and RAC1^V12^ only)

**NE** =[1 0 0] (score: 0; no effect: PGN and RAC1^V12^ not similar to RAS^V12^)

Specificity scores noted above were assigned to candidates closest to the indicated profiles. Candidates with low correlation values (-0.5 < *r <* 0.5) were assigned a score of 1.

Total mRNA nuclear export scores exceeding 4 times that of the *GFP* dsRNA controls were assigned a score of 1.

For assessment of effects on *pMet* promoter activity, *x* corresponds to data from screens # 1, 7, 10, 8, 12 and pMet-GFP (1. RAS^V12^, 7. *Gap1/ Nf1* dsRNA, 10. Insulin, 8. EGFR SPI, 12. RAC1^V12^ and the pMet-GFP screen conducted using secondary probes). The pMet-RAC1^V12^ and pMet-GFP screens were included as indicators of non-specific modulation of pMet (in processes not related to MAPK signaling). The Insulin and *GAP* RNAi screens were included as indicators of specific modulation of MAPK (in a context not dependent on pMet-induced stimulation of pathway activity). Correlation with 4 possible profiles was assessed:

**pMet** =[1 0 0 1 1 1] (score: 2; modulates pMet, but not pMet-independent assays)

**All** =[1 1 1 1 1 1] (score: 1; similar variation in all assays)

**none** =[1 0 0 0 0 0] (score: 0; no modulation of the other assays)

**non-pMet** =[1 1 1 0 0 0] (score: 0; modulates pMet-independent, but not pMet assays)

As for the JNK test, specificity scores noted above were assigned to candidates closest to the indicated profiles. Candidates with low correlation values (-0.5 < *r <* 0.5) were assigned a score of 1. Notably, the epistasis positioning in either the RAS-RAF or RAF-MEK interval is also an indication of the lack of non-specific effect on *pMet* expression (because positioning in these intervals implies lack of effect on *pMet* expression of either RAF and/or MEK). To reflect this, specificity scores were corrected as follows: -2 for RAS-RAF candidates and -1 for RAF-MEK, while candidates. The final *pMet* specificity score could not be lower than 0.

Hit occurrence in previously published *Drosophila* RNAi screens was evaluated using data from FlyMine. The number of screens in which the candidate was also identified was directly added to the specificity score. As a reference, RAS/MAPK components were identified in an average of 4 previous RNAi screens. On the other hand, some splicing factors and ribosome components were hits in over 10 previous screens.

Finally, the effects on MAPK, RAS, CNK and AKT levels were also used to assess specificity. The effects on protein levels in the western blots shown in Figure S4 were scored visually by three separate evaluators. Specificity scores were attributed as follows: No effect on protein levels: 0; depletion of two or more proteins: 1 (weak), 2 (medium), 3 (strong); and depletion observed for a single MAPK pathway protein: -1.

Global specificity scores were defined as follows: high specificity (score ≤ 5) medium specificity hits (5 < score ≤ 8) and low specificity (score > 8). The final specificity score as well as a summary of the different specificity assessments is presented in Table S5.

### Protein Interaction Network

We used the DroID[14] Cytoscape[15] plugin to access *Drosophila* genetic interaction data as well as protein-protein interaction data for *Drosophila* and inferred interactions from yeast, worm and human orthologs. The combined network was created by combining the different homolog networks using the Advanced Network Merge Cytoscape plugin.

### Additional Fly Genetics and Immunohistochemistry Information

Homozygous *Cka* mutant clones were generated using the *flp-FRT* technique[16]. Pupal eye discs were prepared as described in[17] and stained with DAPI, mouse anti-prospero (1/100, DSHB) and rat anti-Elav (1/1000; DSHB).

### Larval Hemocyte Assay

*Ras^V12^* is known to induce overproliferation of larval hemocytes[18]. We used *Hemolectin*-GAL4 (*Hml*-GAL4) to drive expression of *Ras^V12^* in larval hemocytes. RNAi hairpin and GFP constructs were also expressed under the control of the UAS promoter to knockdown candidate genes and mark hemocytes with GFP. Two different fly lines containing *UAS-Ras^V12^* (on either chromosome 2 or 3) and *UAS-lacZ* (used in lieu of an RNAi contruct) were used as positive controls for increased larval hemocyte proliferation. These two controls were needed as the UAS-RNAi constructs were located on either chromosome 2 or 3, and *UAS-Ras^V12^* was added to the chromosome not containing an RNAi construct.

Extraction and scoring of hemocytes was conducted as follows: the hemolymph from 3^rd^ instar larvae was extracted and transferred to Schneider medium in 384-well cell culture plates (Greiner). Each well contained the extracted hemolymph of one larva. Fixed and DAPI-stained cells were then counted using automated microscopy on an Operetta high content imaging system (PerkinElmer). Segmentation and signal quantification was performed using Harmony® software (PerkinElmer). Two separate experiments were conducted, each with at least three biological replicates per condition tested. RNAi were also tested alone (without *RasV12*) to verify if they impacted cell proliferation. None of the RNAi tested (*gfzf, CG4936, Prp19, Caper, CG1603, mapk*) appeared to alter hemocyte counts (not shown) although hemocyte counts were low in larvae not expressing *Ras^V12^* making these results less robust than our experiment with *Ras^V12^* expressing hemocytes.

### Cell Lines and Plasmids

*Drosophila* S2 cells were cultured at 27°C in Schneider medium (Invitrogen) supplemented with 10% fetal bovine serum. S2 cell lines stably transfected with the following constructs were used to conduct RNAi screening assays: *pMet-Ras^V12^*[19], *pMet-raf^ED^*[20], *pMet-mek^EE^*[21], *pHS-sev^S11^*[22], *pMet-EGFR* (a gift from N. Perrimon), *pMet-Rac1^V12^*[23]. *pMet-raf^CT^* and *pMet-raf^EDCT^* respectively encode N-terminal deleted (2-371) RAF made from *pMet-RAF* and *pMet-RAF^ED^*[20]. *pMet-GFP* was made by inserting an *EGFP* cDNA in the *pMet* vector[22].

To construct tagged forms of CKA, STRIP, SLMAP, FGOP2 and MOB4, the respective cDNAs were amplified by PCR with a forward primer containing the desired epitope tag and cloned into the *pMet* vector. For the GFP fusion to FGOP2, the tag was removed from the STag-FGOP2 construct and replaced with a cDNA encoding GFP. In the case of CKA, MOB4, and FGOP2, the tags were fused to the first amino acid following the initiator methionine of the only predicted ORF. For SLMAP, the HSV epitope was fused to the valine at position two of the 897 amino acid ORF (CG17494-PA). For STRIP, the tags were fused to the leucine at position three of the 882 amino acid ORF (CG11526-PA). All constructs were verified by sequencing.

### Protein analysis

For western blot analyses of S2 cell lysates, cells were lysed in cold lysis buffer (20 mM Tris, pH 8.0, 137 mM NaCl, 10% glycerol, 1% Igepal CA-630, 1 mM EDTA) supplemented with 1X phosphatase inhibitor cocktail (Sigma #P2850), 10 μg/mL each aprotinin and leupeptin, and 1 mM PMSF. Lysates were then clarified by centrifugation at 10,000 x g. Protein samples were resolved by electrophoresis on 8 – 12% SDS-polyacrylamide gels and transferred onto nitrocellulose membranes. Specific Drosophila proteins were immunodetected using the following antibodies: anti-CNK (1:5; Douziech et al., 2003); anti-RAS (1:10; Douziech et al., 2003); anti-RAF (1:5000; Douziech et al., 2003); anti-MEK (1:1000; Cell Signaling #9122); anti-MAPK (1:2000; Chemicon #AB3053); anti-JNK (1:2000; Santa Cruz #SC-571); anti-AKT (1:2000; Cell Signaling #9272); anti-Actin (1:2000; Chemicon #MAB1501); anti-PTP-ER (1:10) is a mouse monoclonal antibody that was kindly provided by G.M. Rubin.

For STRIPAK protein expression, S2 cells were transfected with plasmids using Effectene reagent (QIAGEN)., CuSO_4_ (0.7 mM) was added to the media 24 hours after transfection To induce protein expression. Cells were harvested 36 hours post-induction and lysed as indicated above. For immunoprecipitation of epitope-tagged proteins, lysates were incubated with either 0.1 mL anti-HA 12CA5 hybridoma supernatant or 1.5 μg goat polyclonal anti-HSV (Abcam #ab19354) for 4 hours at 4°C in the presence of 20 μL Protein A/G PLUS-Agarose (Santa Cruz). The immunoprecipitates were collected by centrifugation (1000 × g for 2 minutes at 4°C), washed three times with 1 mL cold lysis buffer, and solubilized in Laemmli Buffer. Immunoprecipitates and cell lysates (150 μg total protein) were fractionated on 8% SDS-PAGE gels and transferred to nitrocellulose membranes. The membranes were immunoblotted with anti-STag (GenScript #A00625) at 1:5000 in TBST/5% milk, anti-HSV (GenScript #A00624) at 1:5000 in TBST/5% milk, anti-GFP (Santa Cruz #sc-8334) at 1:1000 in TBST/5% milk, or anti-HA 12CA5 hybridoma supernatant at 1:10 in TBST/5% milk. Antigens were revealed by ECL with HRP-conjugated secondary antibodies.

## SUPPLEMENTARY REFERENCES

1. Ashton-Beaucage D, Udell CM, Lavoie H, Baril C, Lefrancois M, et al. (2010) The exon junction complex controls the splicing of MAPK and other long intron-containing transcripts in Drosophila. Cell 143: 251-262.

2. Roignant JY, Treisman JE (2010) Exon junction complex subunits are required to splice Drosophila MAP kinase, a large heterochromatic gene. Cell 143: 238-250.

3. Barbosa I, Haque N, Fiorini F, Barrandon C, Tomasetto C, et al. (2012) Human CWC22 escorts the helicase eIF4AIII to spliceosomes and promotes exon junction complex assembly. Nat Struct Mol Biol 19: 983-990.

4. Alexandrov A, Colognori D, Shu MD, Steitz JA (2012) Human spliceosomal protein CWC22 plays a role in coupling splicing to exon junction complex deposition and nonsense-mediated decay. Proc Natl Acad Sci U S A 109: 21313-21318.

5. Steckelberg AL, Boehm V, Gromadzka AM, Gehring NH (2012) CWC22 connects pre-mRNA splicing and exon junction complex assembly. Cell Rep 2: 454-461.

6. Singh G, Kucukural A, Cenik C, Leszyk JD, Shaffer SA, et al. (2012) The cellular EJC interactome reveals higher-order mRNP structure and an EJC-SR protein nexus. Cell 151: 750-764.

7. Coelho CM, Kolevski B, Walker CD, Lavagi I, Shaw T, et al. (2005) A genetic screen for dominant modifiers of a small-wing phenotype in Drosophila melanogaster identifies proteins involved in splicing and translation. Genetics 171: 597-614.

8. Goshima G, Wollman R, Goodwin SS, Zhang N, Scholey JM, et al. (2007) Genes required for mitotic spindle assembly in Drosophila S2 cells. Science 316: 417-421.

9. Zhang JH, Chung TD, Oldenburg KR (1999) A Simple Statistical Parameter for Use in Evaluation and Validation of High Throughput Screening Assays. J Biomol Screen 4: 67-73.

10. Marr MT, 2nd, Isogai Y, Wright KJ, Tjian R (2006) Coactivator cross-talk specifies transcriptional output. Genes Dev 20: 1458-1469.

11. Horn T, Boutros M (2010) E-RNAi: a web application for the multi-species design of RNAi reagents--2010 update. Nucleic Acids Res 38: W332-339.

12. Herold A, Klymenko T, Izaurralde E (2001) NXF1/p15 heterodimers are essential for mRNA nuclear export in Drosophila. RNA 7: 1768-1780.

13. Saeed AI, Bhagabati NK, Braisted JC, Liang W, Sharov V, et al. (2006) TM4 microarray software suite. Methods Enzymol 411: 134-193.

14. Murali T, Pacifico S, Yu J, Guest S, Roberts GG, 3rd, et al. (2011) DroID 2011: a comprehensive, integrated resource for protein, transcription factor, RNA and gene interactions for Drosophila. Nucleic Acids Res 39: D736-743.

15. Smoot ME, Ono K, Ruscheinski J, Wang PL, Ideker T (2011) Cytoscape 2.8: new features for data integration and network visualization. Bioinformatics 27: 431-432.

16. Xu T, Rubin GM (1993) Analysis of genetic mosaics in developing and adult Drosophila tissues. Development 117: 1223-1237.

17. Baril C, Therrien M (2006) Alphabet, a Ser/Thr phosphatase of the protein phosphatase 2C family, negatively regulates RAS/MAPK signaling in Drosophila. Dev Biol 294: 232-245.

18. Asha H, Nagy I, Kovacs G, Stetson D, Ando I, et al. (2003) Analysis of Ras-induced overproliferation in Drosophila hemocytes. Genetics 163: 203-215.

19. Therrien M, Wong AM, Kwan E, Rubin GM (1999) Functional analysis of CNK in RAS signaling. Proc Natl Acad Sci U S A 96: 13259-13263.

20. Douziech M, Sahmi M, Laberge G, Therrien M (2006) A KSR/CNK complex mediated by HYP, a novel SAM domain-containing protein, regulates RAS-dependent RAF activation in Drosophila. Genes Dev 20: 807-819.

21. Douziech M, Roy F, Laberge G, Lefrancois M, Armengod AV, et al. (2003) Bimodal regulation of RAF by CNK in Drosophila. Embo J 22: 5068-5078.

22. Therrien M, Wong AM, Rubin GM (1998) CNK, a RAF-binding multidomain protein required for RAS signaling. Cell 95: 343-353.

23. Baril C, Sahmi M, Ashton-Beaucage D, Stronach B, Therrien M (2009) The PP2C Alphabet Is a Negative Regulator of Stress-Activated Protein Kinase Signaling in Drosophila. Genetics 181: 567-579.
